# Plastic degradation by enzymes from uncultured deep sea microorganisms

**DOI:** 10.1093/ismejo/wraf068

**Published:** 2025-11-10

**Authors:** Daniel J Acosta, Daryl R Barth, Julie Bondy, Kathryn E Appler, Valerie De Anda, Phuoc H T Ngo, Hal S Alper, Brett J Baker, Edward M Marcotte, Andrew D Ellington

**Affiliations:** Department of Molecular Biosciences, The University of Texas at Austin, Austin, TX 78712, United States; Department of Molecular Biosciences, The University of Texas at Austin, Austin, TX 78712, United States; Department of Molecular Biosciences, The University of Texas at Austin, Austin, TX 78712, United States; Department of Marine Science, Marine Science Institute, The University of Texas at Austin, Port Aransas, TX 78373, United States; Department of Marine Science, Marine Science Institute, The University of Texas at Austin, Port Aransas, TX 78373, United States; Department of Integrative Biology, The University of Texas at Austin, Austin, TX 78712, United States; Department of Microbiology and Cell Sciences, Fort Lauderdale Research and Education Center, University of Florida, Fort Lauderdale, FL 33314, United States; Department of Chemistry, The University of Texas at Austin, Austin, TX 78712, United States; Department of Molecular Biosciences, The University of Texas at Austin, Austin, TX 78712, United States; McKetta Department of Chemical Engineering, The University of Texas at Austin, Austin, TX 78712, United States; Department of Marine Science, Marine Science Institute, The University of Texas at Austin, Port Aransas, TX 78373, United States; Department of Integrative Biology, The University of Texas at Austin, Austin, TX 78712, United States; Department of Molecular Biosciences, The University of Texas at Austin, Austin, TX 78712, United States; Department of Molecular Biosciences, The University of Texas at Austin, Austin, TX 78712, United States

**Keywords:** metagenomics, archaea, polyethylene terephthalate, plastic degradation, bioprospecting

## Abstract

Polyethylene terephthalate (PET)-hydrolyzing enzymes (PETases) are a recently discovered enzyme class capable of plastic degradation. PETases are commonly identified in bacteria; however, pipelines for discovery are often biased to recover highly similar enzymes. Here, we searched metagenomic data from hydrothermally impacted deep sea sediments in the Guaymas Basin (Gulf of California) for PETases. A broad diversity of potential proteins were identified and 22 were selected based on their potential thermal stability and phylogenetic novelty. Heterologous expression and functional analysis of these candidate PETases revealed three candidates capable of depolymerizing PET or its byproducts. One is a PETase from a Bathyarchaeia archaeon (dubbed GuaPA, for Guaymas PETase Archaeal) and two bishydroxyethylene terephthalate-hydrolyzing enzymes (BHETases) from uncultured bacteria, Poribacteria, and Thermotogota. GuaPA is the first archaeal PETase discovered that is able to depolymerize PET films and originates from a specific enzyme class which has endowed it with predicted novel structural features. Within 48 h, GuaPA released ~3–5 mM of terephthalic acid and mono-(2-hydroxyethyl) terephthalate from low crystallinity PET. PET co-hydrolysis containing GuaPA and one of the newly discovered BHETases further improves the hydrolysis of untreated PET film by 68%. Genomic analysis of the PETase- and BHETase-encoding microorganisms reveals that they likely metabolize the products of enzymatic PET depolymerization, suggesting an ecological role in utilizing anthropogenic carbon sources. Our analysis reveals a previously uncharacterized ability of these uncultured microorganisms to catabolize PET, suggesting that the deep ocean is a potential reservoir of biocatalysts for the depolymerization of plastic waste.

## Introduction

In 2021 the world produced over 390 million metric tons of plastic, 6.2% of which was poly(ethylene terephthalate) (PET) [1]. Plastic production continues to increase, translating to more than 25 million metric tons of PET produced each year with a sizable amount going into single-use packaging. Polyethylene terephthalate-hydrolyzing enzymes (PETases) are a class of enzymes, which can depolymerize the PET polymer down to its monomers of terephthalic acid (TPA) and ethylene glycol (EG) with some incomplete depolymerization products such as monohydroxy ethylene terephthalate (MHET) and bishydroxyethylene terephthalate (BHET) [2, 3]. The TPA released by enzymatic PET depolymerization can be recycled to make new PET and all of the depolymerization products can be used in microbial metabolism for bioremediation or be upcycled into new molecules [4–6].

Efforts to discover more efficient PETases have resulted in 100+ biochemically characterized wild-type enzymes, some of which have been applied to industrial enzymatic recycling applications [4, 7–9]. Most PETases have been isolated from anthropogenic sources, such as compost and wastewater, or mined from large sequence databases via high levels of similarity to enzymes which are already well characterized [10, 11]. The presence of enzymes in the environment capable of depolymerizing polymeric PET is a strong indicator that the organisms in that environment are also capable of depolymerization of postconsumer PET plastics. In contrast, enzymes that hydrolyze phthalic acid esters, or PET oligomers may only indicate consumption of plastic leachates and not polymer depolymerization.

The Guaymas Basin (GB), a marginal rift basin located ~2000 m below sea level in the Gulf of California, is a hotspot for hydrothermally generated hydrocarbons [12] making it an ideal environment to search for novel petroleum-based polymer degrading enzymes. Previous research found a PETase (PET46) in an archaeal metagenome assembled genome (MAG) from this site [13]. This PETase was only able to depolymerize ultrafine powders or a PET trimer and could not depolymerize films or other larger substrates representative of postconsumer PET products. The enzyme was found to have an optimal reaction temperature of 60°C and a melting temperature of 84°C, which are suited for PET depolymerization. Previous studies have used bioinformatic strategies such as the employment of hidden Markov models (HMMs) to screen metagenomic databases [14,15]. By using the sequences of known PETases, it is possible to construct a profile of the amino acid sequence of a PETase and use that to predict new putative PETases from metagenomic data. We recently obtained massive high-throughput metagenomic sequence data from five sediment cores spanning various thermal gradients harvested from four sites of the basin. Here we investigate the presence of PETases and other PET active enzymes from hydrothermally impacted sediments in GB and experimentally validate novel enzymes belonging to the globally distributed archaea, Bathyarchaeia, and other uncultured bacteria.

## Materials and methods

### Metagenomic sequencing, assembly, and binning

Sequencing through *de novo* binning has been previously described in detail for these samples [16]. Briefly, GB sediment push cores were collected by the human-operated vessel Alvin and research vessel Atlantis in November 2018 (AT42-05). Onshore, we extracted DNA for each core subsection across four sites. Library preparation and short-read sequencing was conducted by North Carolina State Genomics Sciences Laboratory (NovaSeq S4 [Illumina]) and University of Delaware (NextSeq [Illumina]). After initial quality control, reads were interleaved and assembled with BBTools Reformat v38.18 [17] and MEGAHIT v1.2.9 [18], respectively. We used assembled scaffolds >2.5 kbp for bioprospecting plastic-degrading enzymes and binning via MaxBin v2.2.7 [19], CONCOCT [20], and MetaBAT [21] via MetaWRAP v1.3.2 [22]. DAS Tools v1.1.2-2 provided a non-redundant set of bins for further analysis [23].

The archaeal PETase (GuaPA) encoding MAG was obtained from a southern mat mound (Latitude: 27°00.37′ N; Longitude: 111°24.57′ W and 2007.2 m below sea level [mbsl]), 15–20 cm deep with a thermal gradient ~20–38°C. The uncultured bacterial MAGs were extracted from Aceto Balsamico microbial mat (Latitude: 27°00.47′ N; Longitude: 111°24.44′ W and 2008.87 mbsl) and Cathedral Hill (Latitude: 27°00.67′ N; Longitude: 111°24.26′ W and 2012 mbsl), respectively. The Aceto Balsamico site (Marker 14) was previously named for high acetate and methane concentrations, supporting a diverse community [24,25]. The genome encoding the first BHETase (B1) was from an Aceto Balsamico site, 20–30 cm deep and had temperature readings between ~74.1 and 115°C. Whereas, the genome encoding the second BHETase (B4) was from a Cathedral Hill site 5–13 cm deep core with temperature of 89°C measured at a 25 cm core depth.

### Computational bioprospecting

Using Prodigal v2.6.3, we predicted all the open reading frames of the GB 2.5 kb scaffolds (GE3) described previously [16]. These 7 782 778 million proteins became the database used to search for potential enzymes. To develop the search criteria, we used the PlasticDB [26], a database of known plastic-degrading enzymes, as the input enzymes. Using MMseqs2 [27], we clustered proteins listed under PET in PlasticDB by 30% sequence similarity, generating three clusters of PET-degrading enzymes. The largest cluster contained IsPETase, Leaf Compost Cutinase (LCC), and 55 other enzymes, whereas the other two clusters were sparse, containing three and two sequences, respectively. We computed a multiple sequence alignment (MSA) of proteins in the largest cluster using MUSCLE v3.8.1551 [28], then generated HMM profiles from the resulting MSA using the HMMER v3.3 software suite hmmbuild function. Using these HMM models, we searched the previously generated GB protein database for hits ≤1E-5, identifying 362 unique candidate enzymes. These hits were aggregated and filtered using custom python scripts available at github.com/marcottelab/GuaPA. Then, the candidate enzymes were clustered by 40% sequence similarity using CD-HIT v4.8.1 [29,30]. From the 32 resulting clusters, candidates with the lowest *E*-values from the initial HMM search were selected, and their protein structures were predicted using ESMFold [31], a large language model-based sequence-to-structure predictor that has previously been used for the efficient prediction of millions of metagenomic protein structures. Supplementary Fig. S1 depicts a phylogenetic tree showing the distribution of the candidates. Foldseek was then used to compare the structures of these candidates to the canonical enzymes IsPETase and LCC. The best 22 structural matches were then selected to move forward.

Prior to synthetic expression, the deep learning model TMBed [32], the software packages SignalP 6.0 [33], and Unbiased Organism-agnostic Signal Peptide Network (USPNet) [34] were applied to the 22 chosen sequences to predict signal peptides. Manual comparison of the output from these three methods determined the exact sequences to remove from these candidates. The predicted signal peptides extracted from the N-termini of the 22 candidate proteins are provided in Supplementary Fig. S2 and the removed sequence on GuaPA is highlighted in Supplementary Fig. S3.

### Media and microorganisms

LB Broth Miller and LB Agar Miller were purchased from Fisher BioReagents. Terrific Broth was purchased from Sigma-Aldrich. *Escherichia coli* strains BL21 (DE3) and DH5α chemically competent cells were purchased from New England BioLabs.

### Cloning and protein expression

The 22 putative PETases (Supplementary Table S1) were ordered as gene fragments from TWIST Biosciences (South San Francisco, CA, USA) codon optimized for *E. coli* and flanked by BsaI cut sites. The gene fragments were cloned into pET28α which was amplified with primers to incorporate BsaI cut sites which inserted the putative PETase genes in-frame with the NcoI Start codon and 6x-C-terminal His tag present in the original vector. DH5α chemically competent cells were transformed with the assemblies, and transformants were selected on LB agar + 50 μg/ml of kanamycin. For protein expression, BL21 (DE3) chemically competent cells were freshly transformed with sequence verified plasmid. Single colonies were used to inoculate overnight cultures of LB + kanamycin, which were used to inoculate flask cultures of Terrific Broth + kanamycin at a 1:100 ratio of medium to culture. Flask cultures maintaining a medium-to-flask volume ratio of 1:5 were grown at 37°C and 220 RPM until an OD_600_ 0.4–0.6 was reached (in ~2–4 h). Flasks were placed in a 4°C cold room for 15 min, then induced with IPTG to a final concentration of 1 mM and allowed to shake at 220 RPM and 18°C for 18 h or overnight.

### Protein purification

Cells, which had been induced and allowed to express protein for 18 h or overnight, were harvested via centrifugation at 6000 × g at 4°C for 25 min. The cell pellets were resuspended in 30 ml of resuspension/lysis buffer (50 mM phosphate buffer pH 7.5, 300 mM NaCl, 20 mM imidazole, 0.1% Igepal CO-630 (Sigma-Aldrich), and 5 mM MgSO_4_. Resuspended cells were lysed via sonication using 40% amplitude and 4 min of sonication time with 1 s on 4 s off. The cell lysate was clarified by ultracentrifugation at 4°C for 30 min at 35 000 × g. The clarified cell lysate was applied to a disposable 10 ml gravity column with a 1–2 ml resin bed (column volume (cv)) of Ni-NTA resin (Thermo) which had been equilibrated with 20 cv of equilibration buffer (50 mM phosphate buffer, pH 7.5, 300 mM NaCl, and 20 mM imidazole). The sample column was washed with 20 cv equilibration buffer and 5 cv wash buffer (50 mM phosphate buffer, pH 7.5, 300 mM NaCl, and 50 mM imidazole). Protein was eluted using 5 ml of elution buffer (50 mM phosphate buffer, pH 7.5, 300 mM NaCl, and 250 mM imidazole). The eluted protein was concentrated with an Amicon Ultra-15 Centrifugal Filter MWCO 10 000 spin filter and desalted by adding 15 ml of PBS to the concentrated sample and concentrating again. The concentrated, desalted protein was stored at 4°C for up to 2 weeks or mixed 1:1 with glycerol and stored at −20°C.

### Enzyme assays

Enzymes concentrations were determined using Bradford assays. Enzyme activity was assayed using 10 mM BHET or a 6 mm hole punch of PET film, either beancake film or Goodfellow low crystallinity PET film as specified. Reactions were performed in a total of 600 μl using 100 mM potassium phosphate buffer pH 8 in protein low-bind tubes unless otherwise specified. BHET reactions were performed at 30°C for 24 h. BHET reactions were also performed after pre-incubating the tube with no BHET at 70°C for an hour and adding BHET once the tube had cooled. For PET assays, tubes were incubated for 48 h at either 30°C or 60°C unless otherwise specified. All reactions were performed in triplicate and with substrate and buffer only as a negative control. To stop the reactions, the entire reactions or aliquots of the reactions were mixed 1:1 with DMSO [35] (Supplementary Fig. S4).

### High-performance liquid chromatography quantification of polyethylene terephthalate and bishydroxyethylene terephthalate hydrolysis

To quantify hydrolysis of PET and BHET, samples from enzyme assays were filtered using 0.2 μM syringe or 96-well plate centrifuge filters. These samples were then measured using a Vanquish HPLC system (Thermo Fisher Scientific) with a 260 nM UV detector. The mobile phase buffers consisted of 0.1% formic acid in water or 0.1% formic acid in acetonitrile over the course of 30 min with a fixed flow rate of 0.8 ml/min. The mobile phase used the following conditions: a solvent gradient of 1%–5% organic (vol/vol) for 5 min, a solvent gradient of 5%–100% organic (vol/vol) for 8 min, 100% organic solvent for 10 min, a solvent gradient of 100%–5% organic (vol/vol) for 2 min, and finally a solvent mix of 5% organic (vol/vol) for 5 min. Two-standard curves of BHET, MHET, and TPA were prepared for high and low ranges of detection in the linear ranges of 200 μM to 1.2 mM and 1.2 mM to 12 mM.

For BHET hydrolysis assays, the quantity of MHET and TPA was measured by subtracting the calculated mean quantity of TPA and MHET in the enzyme free negative control reactions from the calculated concentration of MHET and TPA in the samples.

### Polyethylene terephthalate catabolism pathway structural homology search

We obtained protein sequences for monomer catabolism from UniProt [36] and searched the MAGs containing B1, B4, and GuaPA to determine pathway completeness (*E*-value ≤ 1e-5). In parallel, we downloaded the predicted protein structures of the known enzymes necessary for the metabolic process from the AlphaFold Database [37] and used ESMFold [31] to predict the structures of the proteins present in the MAGs. We then used Foldseek with an *E*-value cutoff of 1e-5 to search the MAGs for any structural similarity.

### Polyethylene terephthalate-hydrolyzing enzymes phylogenomic analyses

Database identifiers for 105 known PETase enzymes were downloaded from the PAZy database [15] and used to download the corresponding amino acid sequence from GenBank, UniProt, or MGnify. The sequences were then combined with candidate PETase enzymes from the GB samples. A MSA was computed via MAFFT v7.453 [38,39] and trimmed with ClipKIT v1.3.0 [40], and the phylogenetic tree was computed with IQ-TREE v1.6.12 [41]. The position of the 22 GB candidate enzymes in a more global phylogenetic tree was further determined by searching for homologs in the NCBI non-redundant database [42] (ca. September, 2024), retaining the top 50 hits for each sequence above an *E*-value of 1E-5. Duplicate sequences were removed, the sequences realigned with MAFFT auto v7.526 [38], and 50% of the gaps masked with Geneious Prime 2023.2.1 [43]. This overall dataset was then used to infer a phylogeny using IQ-TREE v2.0.7 [44]. The best-fit model WAG + R10 was chosen according to Bayesian information criterion. Phylogenies were visualized with iTOL v6 [45].

### Phylogenetic analyses of polyethylene terephthalate-hydrolyzing enzyme candidate-encoding genomes

GTDB-tk v2.4.0 [46] initially classified the genomes encoding B1, B4, and GuaPA as *Poribacteria*, *Thermotogota*, and *Thermoproteota*, respectively. To verify the predicted taxonomic assignment, we selected publicly available bacterial genomes from (NCBI) belonging to Deinococcota, Synergistetes and Thermotogae (DST) phyla (*Deinococcota*, *Synergistota*, *Lindowbacteria, Spirochaetota, Elusimicrobiota, Desantibacteria, Mcinerneyibacteriota, Fusobacteriota, Poribacteria*) and basal *Gracilicutes* phyla (*Caldisericota, Lithacetigenota, Coprothermobacterota, Bipolaricaulota,* and *Thermotogota*). Archaeal references were chosen from *Thermoproteota* classes (*Bathyarchaeia, Korarchaeia, Methanomethylicia*), orders (*Brockarchaeales, Caldarchaeales, Conexivisphaerales, Gearchaeales, Geothermarchaeales, Marsarchaeales, Nitrososphaerlaes, Panguiarchaeales, Sulfolobales, Thermofilales, Thermoproteales*), and an outgroup genus *Methanobacterium*. We extracted 37 cross-domain markers via the PhyloSift [47] pipeline to infer the phylogenetic placement of the archaeal and bacterial PETase candidate-encoding genomes within the reference set. The resulting concatenated alignments were realigned with MAFFT v7.490 [38,39] (auto) and masked (50% gaps) with Geneious Prime 2023.2.1 [43]. Phylogenetic trees were inferred with IQ-TREE v2.0.7 [44] for the domains separately based on 1000 ultrafast bootstrap inferences and optimized with hill-climbing nearest neighbor interchange. The archaeal and bacterial trees were rooted with *Methanobacterium* and between *Terrabacteria* and *Gracilicutes*, respectively. The resulting phylogenies were visualized with iTOL v6 [45].

## Results

### Identification of polyethylene terephthalate-hydrolyzing enzymes from thermophilic ocean floor microorganisms

Recent studies have identified an increasing number of novel PETase enzymes from a variety of non-marine sources [15]. Given the global spread of plastic waste, we hypothesized that PETases might be found in deep sea microbial sources. We therefore plumbed a genomic catalog from the GB, a hydrothermal vent system in the Gulf of California [16,48] known to provide a wealth of information regarding new phyla and metabolic capabilities [48]. In particular, PETase sequences were mined from previously described samples from the GE3 [16] expedition of November 2018. During this expedition researchers collected sediment cores from ~2000 mbsl using a manned submersible, Alvin. The core samples were frozen at −80°C until DNA was extracted onshore [16] and sequenced using a combination of the NovaSeq S4 and NextSeq (Illumina) platforms, depending on DNA quantity. Protein-coding sequences from this metagenomic dataset were identified and annotated with Prodigal v2.6.3 [49], ultimately generating a set of 7 782 778 unique protein sequences (see Materials and methods).

**Figure 1:**
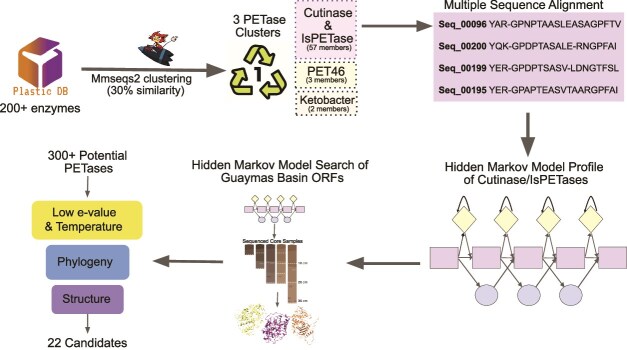
Computational pipeline picturing the process taken for aggregating known plastic degrading sequences, constructing a HMM profile, searching GB metagenomic data and subsequently downselecting potential enzyme candidates.

A HMM profile was built from known plastic-degrading enzymes and used to search the compiled protein sequences (Fig. 1). Some 300 unique putative enzymes were identified and filtered to a top 22 candidates based on their thermophilic potential, as determined by the temperature of the sampling site where the DNAs were acquired, phylogenetic novelty, and structural similarity to known PETases (Supplementary Fig. S1). The majority of candidates chosen had less than 30% sequence identity with more well-known enzymes such as *Ideonella sakaiensis* PETase (IsPETase) and LCC, and at most 65% identity to known PETases listed in the PAZy database [15], with an overall average identity of 27.6% (Supplementary Fig. S5).

### Screening of candidate enzymes on bishydroxyethylene terephthalate and amorphous polyethylene terephthalate

The 22 candidate enzymes were codon optimized, a C-terminal hexa-histidine tag added, and genes were expressed following cloning into pET28a and transformation into *E. coli* BL21 DE3. The putative PETases were purified using a nickel chelate column and protein was obtained from all 22 identified sequences, in amounts ranging from 0.4 to 20 mg/100 ml culture. The proteins were estimated to be >90% pure via polyacrylamide gel electrophoresis (PAGE) (Supplementary Fig. S6).

**Figure 2:**
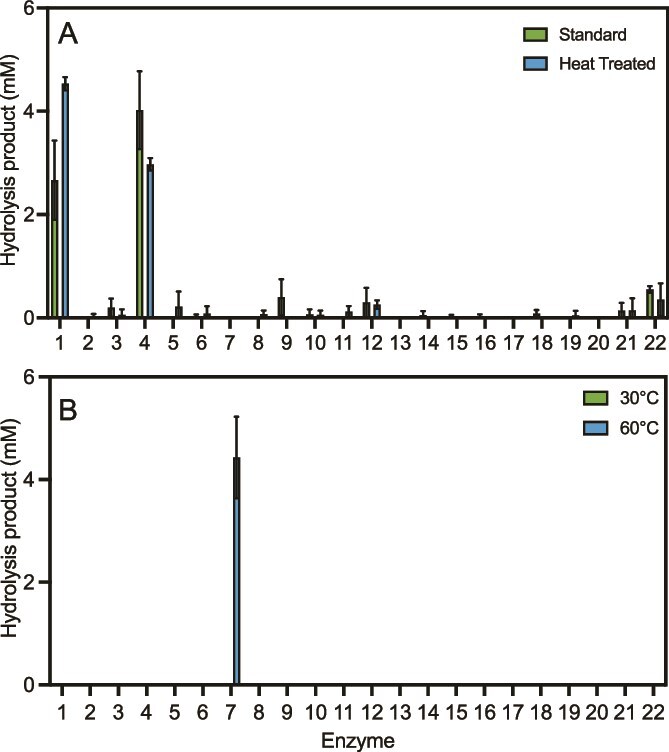
Hydrolysis products of 22 putative PETases using beancake film amorphous PET (A) and BHET (B) as substrates. All reactions were run in 600 μl 100 mM KPB pH 8 with an enzyme concentration of 200 nM using either a 6 mm hole punch of PET film or 10 mM BHET. PET reactions were incubated for 48 h, while BHET reactions were incubated for 24 h.

Candidate enzyme activities were determined by applying enzyme samples to amorphous, beancake film PET (BC-PET), a relatively easy-to-degrade PET film, as described previously [4], and examining plastic breakdown products using high-performance liquid chromatography (HPLC). In an initial screen each of the 22 candidate PETases at 30°C no evidence of PET hydrolysis was observed (Fig. 2A). Knowing that some thermophilic enzymes might prove inactive at mesophilic temperature ranges, the entire PETase candidate list was rescreened on BC-PET at 60°C. Enzyme candidate 7, from *Bathyarchaeia* (Supplementary Fig. S7; GuaPA), was found to hydrolyze BC-PET, releasing a combined 4.4 mM of the depolymerization products TPA, MHET, and BHET after 48 h (Fig. 2A). GuaPA is thus the only archaeal enzyme that has been shown to depolymerize PET film.

To ascertain whether any of the candidate enzymes could also degrade BHET or MHET, all 22 enzymes were further assayed using BHET as a model substrate, initially at 30°C because BHET undergoes significant autohydrolysis at higher temperatures [50]. GuaPA was not able to hydrolyze BHET; however, Guaymas enzymes 1 and 4 (B1 and B4) were found to hydrolyze BHET to yield combined MHET and TPA products (2.7 and 4.0 mM, respectively, Fig. 2B). The production of TPA indicates that both B1 and B4 hydrolyze BHET to MHET and can further hydrolyze MHET to yield TPA. A pre-incubation of the enzymes at 70°C, followed by incubation with BHET at 30°C revealed that both B1 and B4 remained active following the thermal pulse and were still able to hydrolyze BHET (Fig. 2B). Given that the three active enzymes identified from the putative list of PETases appeared to be thermophilic, or at least thermotolerant, we decided to investigate their respective melting temperatures using differential scanning fluorimetry (DSF, the Biotium GloMelt system). The melting temperatures of GuaPA, B1, and B4 were 79, 87, and 80°C, respectively (Supplementary Fig. S8).

### Optimizing polyethylene terephthalate hydrolysis by GuaPA

We sought to optimize GuaPA’s performance. Reaction parameters such as salt concentration, pH, enzyme concentration, and temperature were initially optimized; each was varied independently, using BC-PET as a substrate. The best reaction conditions were 200 mM KPB, pH 8, at 60°C (Supplementary Fig. S9A). The higher salt concentration preferred by GuaPA, 200 mM, compared to other PETases may be related to the enzyme's relatively low isoelectric point 4.9 or its oceanic origin. GuaPA contained what appeared to be a 24 amino acid signal peptide (Supplementary Fig. S3). When expressed with and without this leader the truncated version appeared to perform slightly better, but the differences were not statistically significant (Supplementary Fig. S9B).

We observed that GuaPA was most active at lower enzyme concentrations (100 nM). This contrasts with other PET-degrading enzymes, such as LCC and its engineered variants, which are typically used at micromolar concentrations. There is no loss of activity upon dilution of GuaPA from higher concentration (Supplementary Fig. S10), suggesting that the phenomenon is due to reversible interactions between the enzyme and the plastic or between enzyme molecules.

### Improving polyethylene terephthalate degradation with enzyme cocktails

**Figure 3:**
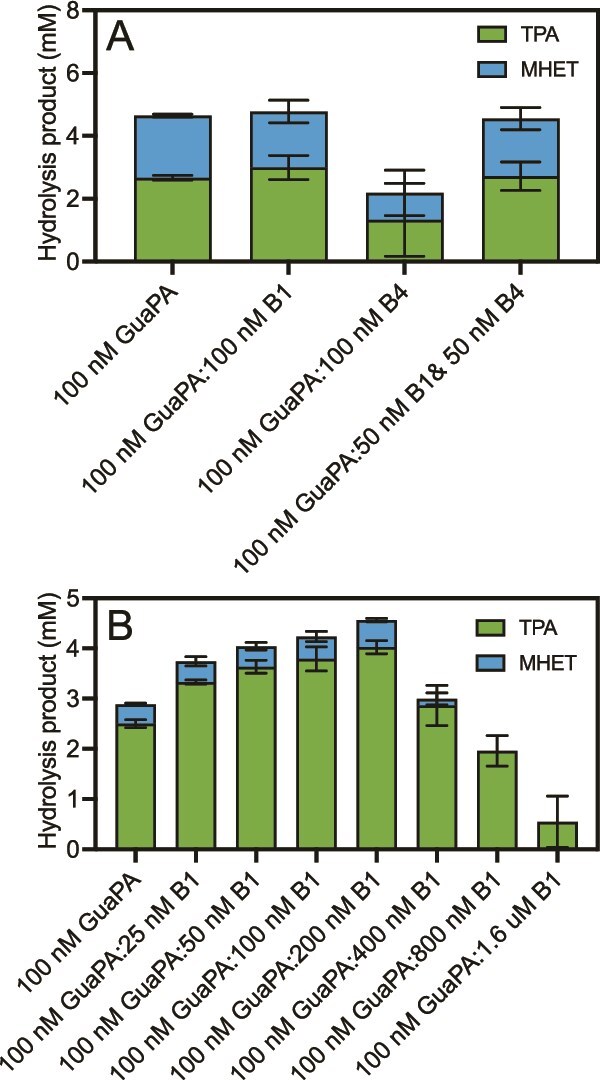
Depolymerization of PET films using two enzyme reactions either. (A) Depolymerization of BC film by GuaPA or combinations of GuaPA and BHETases. (B) Optimization of the ratio of GuaPA and B1 for increasing PET depolymerization of goodfellow low crystallinity PET.

Previous research has shown that the inclusion of BHETases can improve PET hydrolysis by removing BHET and MHET intermediates that can occupy the PETase active site but are not efficiently hydrolyzed [51]. To assess this possibility with our newly discovered enzymes, we combined equimolar amounts of GuaPA and the BHETases B1, B4, or a combination of B1 and B4 (Fig. 3A). B1 or a combination of B1 and B4 showed no difference in hydrolysis, B4 seemed to significantly hinder PET hydrolysis.

To discern if B1 may improve PET hydrolysis with a more crystalline, and thus difficult to depolymerize substrate, B1 assays were carried out with 8% crystalline PET from Goodfellow. Although this is still low crystallinity PET, it is more difficult to degrade than the BC-PET which is 1%–2% crystallinity. On Goodfellow PET, GuaPA depolymerized an average of 2.9 mM of product, which consisted of 86% TPA and 14% MHET over 48 h (Fig. 3B). When combined at a ratio of one molecule of GuaPA to two molecules of B1, maintaining the concentration of GuaPA at 100 nM, hydrolysis is increased by 1.7-fold to a total hydrolysis of 4.6 mM combined TPA and MHET, with 88% of the release product again being TPA. As the proportion of B1 further increases, overall yields begin to fall with TPA eventually becoming 100% of the released product.

With both Goodfellow film and BC PET GuaPA releases depolymerization products not previously seen during the enzymatic depolymerization of PET films, whether by FAST PETase, LCC, or other PETases [4]. LC–MS analysis determined that the products are small oligomers of PET (Supplementary Fig. S11), suggesting that GuaPA may function primarily as an endo-PETase. This is consistent with GuaPA’s inability to hydrolyze BHET (Fig. 2B). This pattern of substrate preference has not been demonstrated among known PETases. These products disappear with the inclusion of B1, suggesting that B1 may be able to degrade PET oligomers larger than BHET.

### Predicted structural properties of GuaPA

**Figure 4:**
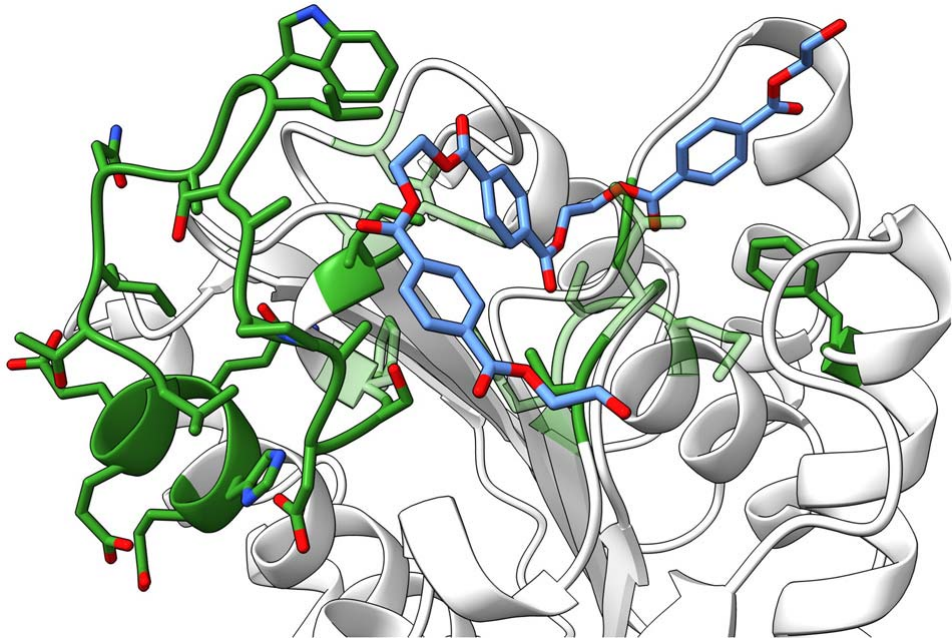
Residue differences in the structure of GuaPA in the context of a PET trimer compared to previously described PETases. Residues which are part of the catalytic triad, Subsite I, Subsite II, the extended loop, and the additional disulfide bond are shown as stick models. Those residues that do not fit into either of the traditional Type I or Type II PETases are opaque whereas those that do are transparent.

**Figure 5:**
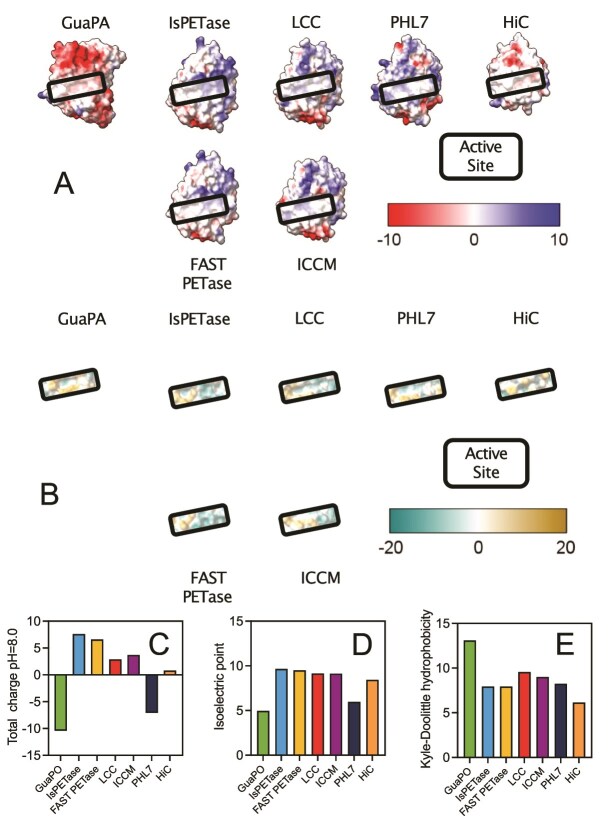
Surface properties of GuaPA compared to IsPETase, FAST PETase, LCC, ICCM, PHL7, and HiC. (A) Surface charge map of the catalytic face of 7 PETases demonstrating the highly negatively charged face of GuaPA in comparison to other PETases. (B) Hydrophobicity map of 7 PETases demonstrating the slightly larger hydrophobic pocket surrounding the active site of GuaPA. The net charge (C), isoelectric point (D), and hydrophobicity (E) of 7 PETases show GuaPA’s unique combination of low charge, low isoelectric point, and higher hydrophobicity.

When an ESMFold [31] model was used to predict the structure of GuaPA it did not fall neatly into the Type I, Type II classification system previously used for categorizing PETases [52], and instead contained distinct structural features that differentiated it from other previously described PETases (Fig. 4). GuaPA is most different from other PETases in its extended loop region: this loop, which is 3–6 amino acids in Type I and Type II enzymes, is 12 amino acids in GuaPA. Docking a PET trimer in the active site of GuaPA using Chai-1 [53] shows that this loop expansion increases the number of hydrophobic amino acids proximal to the PET chain, potentially enlarging the hydrophobic cleft region. This expansion has been shown to be beneficial in other PETases by creating additional hydrophobic interactions that draw the PET chain into the active site [3,52,54]. The surface of GuaPA is very negatively charged (Fig. 5A and C), a property which was previously deemed to be a hindrance to PET hydrolysis [55]. The hypothesis that a negatively charged enzyme or active site might not be able to approach carboxylates on the PET surface was chemically suspect, and the existence of GuaPA suggests that PETases writ large may have a variety of ways in which they can achieve plastics breakdown. Emphasizing such new possibilities, the demonstrated thermostability of GuaPA was attained even in the absence of a disulfide bond, a feature that is unique compared to other thermostable PETases [8,56]. We speculate that in the absence of disulfide bonds GuaPA may be stabilized by a network of π–π interactions. Using the Contacts of Aromatic Residues in Proteins analysis pipeline [57], GuaPA was found to have 33 predicted aromatic–aromatic residue interactions (Supplementary Fig. S12), whereas LCC had only 24 and IsPETase only 13 (Supplementary Fig. S12 and Supplementary Tables S2–S4). This collection of unique structural features makes GuaPA an especially appealing candidate for future enzyme engineering, potentially by incorporating the beneficial attributes of canonical PETases into its structure.

### Predicted metabolism of polyethylene terephthalate derived monomers by enzyme containing metagenomes

**Figure 6:**
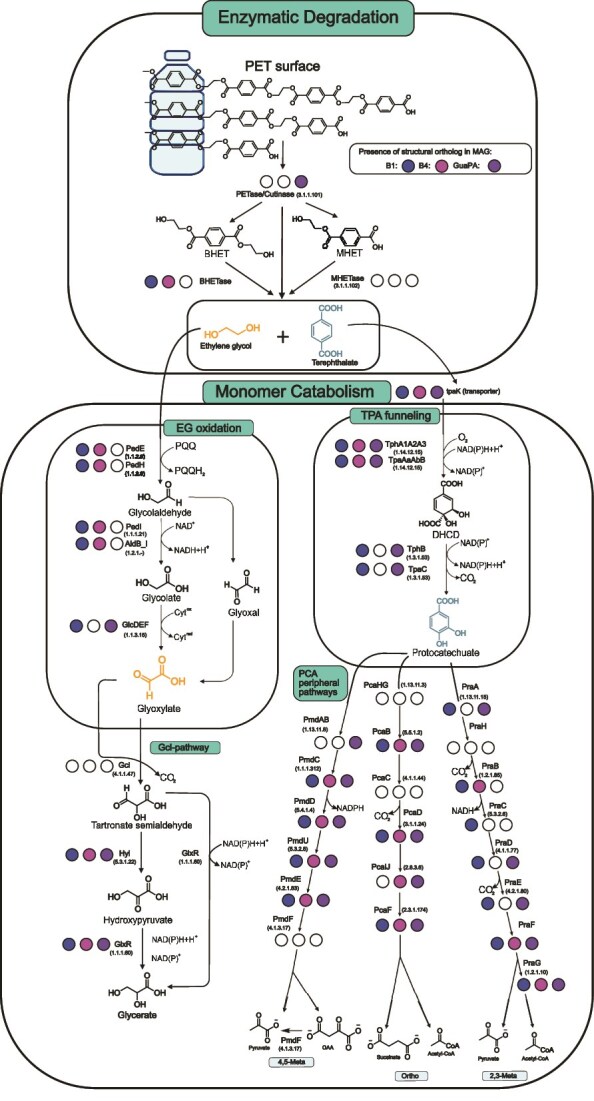
PET degradation and monomer catabolism pathway. Filled in circles represent whether a structural homolog for the given enzyme is present in the organism where B1, B4, or GuaPA resides. EC numbers are listed below each of the named enzymes in parentheses.

The different enzymatic features of GuaPA led us to speculate that the organisms from which it was derived might also have unique PET degradation pathways. Given the ability of B1 and B4 to hydrolyze BHET, we wondered whether the metagenomic samples might also contain pathways capable of metabolizing liberated TPA and EG. The metabolic pathways to fully metabolize PET have been described previously [58], but searching for similar sequences via BLAST did not return homologous catabolic pathways. Nonetheless, many enzymes that might participate in homologous pathways were found using Foldseek structural similarity searches (Zenodo Repository) *Bathyarchaeia* MAG encoding GuaPA contains all of the genes necessary to import PET and degrade it to TPA, and from there to protocatechuate (Fig. 6), as well as pathways for the further integration of TPA into central metabolism via either the 2,3-meta-cleavage or the 4,5-meta-cleavage pathway, similar to other metabolically diverse prokaryotes like *Comamonas testosteroni* [58].

## Discussion

The newly identified thermophilic PETases from deep sea sediments may prove to be excellent candidates for engineering and translation [59], given their already very high thermotolerance, atypical isoelectric point, apparent preference for interior ester bonds, and ability to completely degrade PET to TPA and MHET. For example, GuaPA is significantly more thermostable than IsPETase, and only slightly less thermostable than LCC, one of the natural PETases with the highest reported melting temperatures [4,8,60].

The discovery of this enzyme in deep sea sediments may reveal a bounty of new opportunities for further biomining these sediments, given that computational analyses reveal unique enzyme features (Figs 4 and 5) and potentially unique pathways for the catabolism of PET degradation products (Fig. 6). In the validated PAZy database, there is only one other archaeal PETase (PET46) from an uncultivated GB *Bathyarchaeia* [61]. That said, GuaPA is the only archaeal enzyme that has been shown to depolymerize PET film and has a unique position in the phylogenetic tree of PETases. Additionally, B1 is from an isolate of the phylum *Poribacteria*, which are known commensals of marine sponges [62], and B4 is from the genus *Thermotogota*, which are known to be a part of general macromolecule degrading communities in the GB [63] (Supplementary Fig. S13). Like archaea, these bacteria are less well represented marine microorganisms in plastics degradation enzyme databases.

The identification of PETases from the deep ocean should at some level come as no surprise. Plastic pollution is permeant on the planet, and the deep ocean has been shown to be a major sink, harboring up to 11 million metric tons of plastic [64], a mixture of oceanic plastic from fishing equipment and terrestrial plastic deposited in the deep ocean by currents, with PET, nylon, and polyethylene [65,66] making up large percentages. Plastic degrading enzymes have been catching up with this rich new source of carbon [67]. Because the evolution of plastic degradation is still in its infancy, however, it is likely that there will be many parallel enzymes and pathways invented to supplement organismal growth.

**Figure 7:**
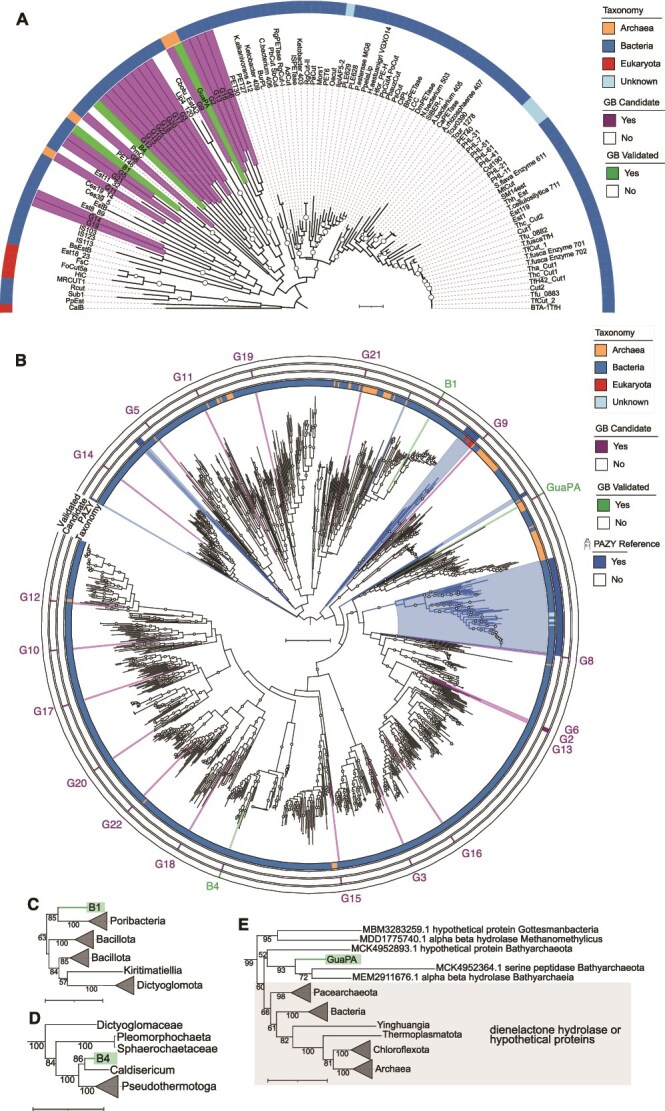
Phylogenetic distribution of candidate and validated Guaymas Basin (GB) PETases. (A) Unrooted phylogenetic tree of five published wild-type PET-hydrolyzing enzymes from the PAZy database along with the 22 GB candidates. The colored blocks indicate the taxonomic group from which the enzymes originate, according to PAZy. We highlighted the unvalidated and the active GB enzymes on PET or BHET. Tree scale is 0.1. (B) To increase phylogenetic support, we added unvalidated non-reference hits from the NCBI non-redundant sequence database for each of the 22 GB candidates, including the three validated enzymes. This expanded analysis includes 94 PAZy reference sequences and 998 blast sequences. We inferred the maximum likelihood phylogeny with IQ-TREE v2.0.7, using the best-fit model WAG + R10 chosen according to Bayesian information criterion. The concentric rings highlight taxonomy, PAZy references, GB candidates, and GB validated enzymes. Ultrafast bootstrap support values ≥90 are shown and midpoint rooted. Insets show sequences clustering with the validated GB enzymes: (C) B1, (D) B4, and (E) GuaPA. Ultrafast bootstrap support values ≥50 are shown for the insets. For (B–E), the tree scale is equivalent to 1.

GuaPA is likely just the tip of the iceberg in terms of identifying PETases and other plastic-degrading enzymes. Phylogenetic analysis of enzymes related to GuaPA identified 93 further archaeal homologs from diverse sources, suggesting the archaeal diversity of PETases remains largely untapped (Fig. 7). Broadly, the origins of GuaPA are far less clear than for other PETases, which are largely derived from cutinases (Fig. 7A and B). GuaPA is found to cluster with alpha/beta hydrolases, a serine peptidase, and dienelactone hydrolases (Fig. 7E). Dienelactone hydrolases have previously been shown to have BHETase-like activity [68]. The identification of new PETases that are not derived from cutinases ultimately tracks with the relative lack of cutin in oceanic environments [69], as opposed to the abundance of cutin in terrestrial plants [70].

The integration of sequence-based discovery methods, structural predictions, and phylogenetic data has unearthed new plastic-degrading enzymes that likely would have been missed by a sequence-only approach. Not only can new PETases be discovered, but also structural homology searches have enabled the identification of new and potentially unique downstream enzymes for PET degradation (Fig. 6). As but one key example, B1 and B4 would not have been found if phylogenetic relatedness had been the sole criteria for selection. The genomic context of the newly discovered PETases may further reveal previously unknown plastic degradation pathways. For instance, the GuaPA genome appears capable of incorporating TPA into central metabolism via a multitude of pathways (Fig. 6), all of which may be repurposed for the next generation of PET biotransformation, with the same being true of the EG incorporation pathways present in the MAGs of B1 and B4 (Fig. 6). Further interrogation of deep sea sediments may thus well continue to turn up entirely new solutions to PET degradation that differ significantly from their terrestrial counterparts, and may yield even more tractable engineering solutions for PET valorization; e.g. the successful combination of GuaPA and B1, enzymes from a *Bathyarchaeia* and *Poribacteria*, respectively, may foreshadow functional syntrophic relationships between GB organisms that could be exploited by creating synthetic pathways for plastic degradation. As another example of how nature can lead biotechnology, combining the metabolic capacities of the GuaPA for TPA and B1 for EG could potentially improve not only the depolymerization of PET (Fig. 3), but also the downstream biotransformation of PET into glycerate and pyruvate, ultimately remediating the plastic while generating useful chemical feedstocks for additional transformations.

## Supplementary Material

Supplemental_1_wraf068

## Data Availability

All sequence data and GB sample information are available at NCBI under BioProject ID PRJNA1112871. All raw data underlying phylogenomic (alignments and resulting phylogenetic trees) and structural analyses (pdb structures) have been deposited into Zenodo (10.5281/zenodo.14262448) along with the MAGs containing B1, B4, and GuaPA. Custom code and python scripts are available at github.com/marcottelab/GuaPA.
