# Supplementary material for: Plastic degradation by enzymes from uncultured deep sea microorganisms
Source: ISME J. 2025 Nov 10;19(1):wraf068. doi: 10.1093/ismejo/wraf068 (PMC12599313; doi:10.1093/ismejo/wraf068)
Supplement: Supplemental_1_wraf068 [file supplemental_1_wraf068.pdf]

Supplementary information 1

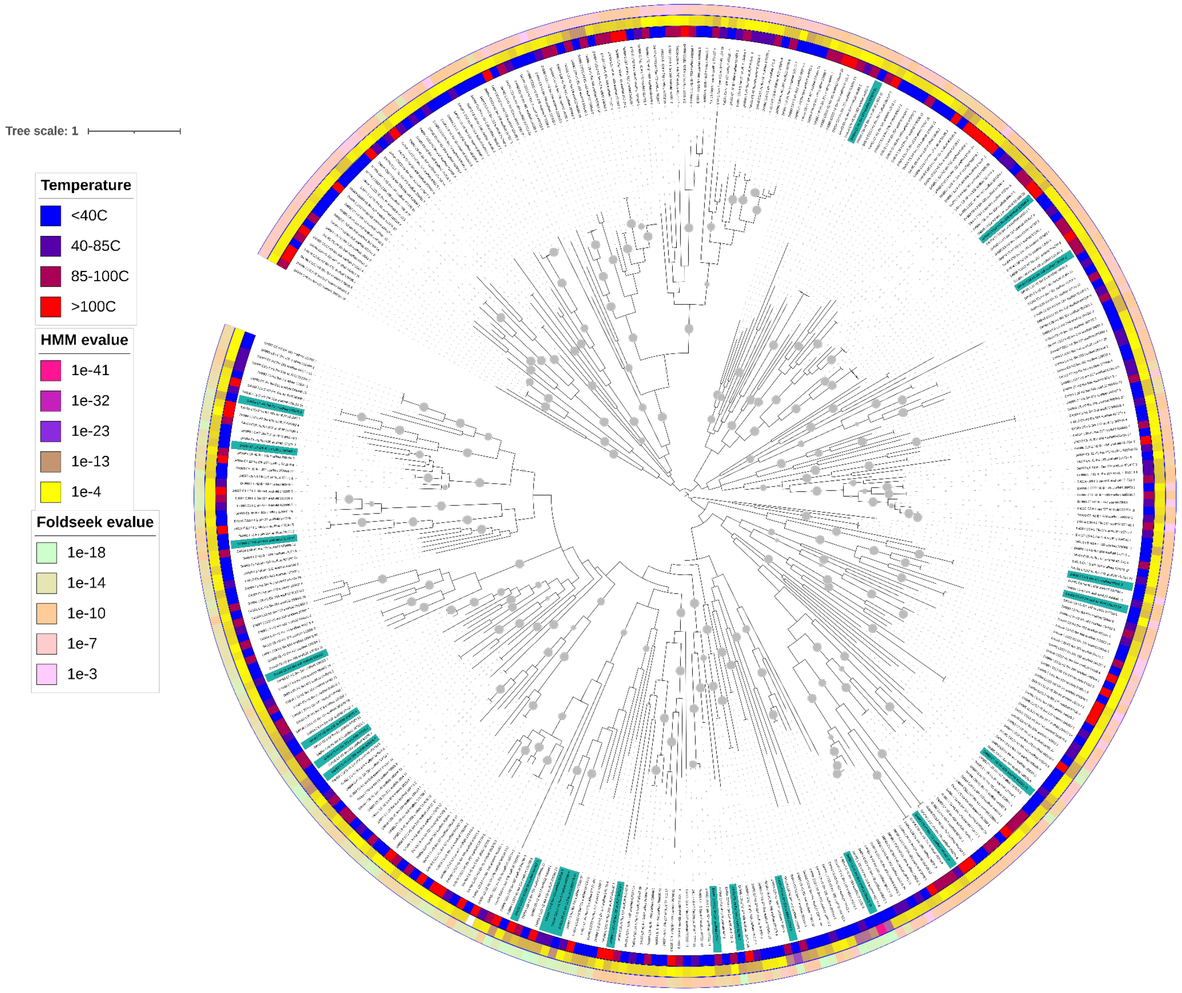

Supplementary Figure 1. Phylogenetic tree depicting the distribution of the 22 candidate enzymes, highlighted in teal, picked among the 360 unique hits from the HMM search conducted on the assemblies. The three concentric colored rings depict, from innermost to outermost, measured temperature of the core from which the enzyme was mined, the e-value from the HMM search, and the e-value from the Foldseek search. The maximum likelihood phylogeny was inferred with IQ-TREE v2.0.7, using the best-fit model WAG+R10 chosen according to Bayesian information criterion (BIC). Ultrafast bootstrap support values >= 95 are shown and midpoint rooted.

>2

MKKTAIYITYILTICTISIFAQSYEMGHRSEIYIDPSRGDRAIPTEIYYPSLTAGDDVPVADPPTGGFPVVAFAHGRMMPWDDYENIWGALAPQGFVVACP...

>4 (B4)

MNSKLLFAILCMLITVVFAFGQNSANVQNEISYDVFDYAQHPMGTIWGDPLPDAPALAARGEYMVGVQTLHLSNPNQTDVINTITSKSTVIYDRPLTVEVW...

>6

MKKLSYIFILCILSLIFLPVFAHSQSYQIGHTSIIYYDANRSNREIPTEIYYPAENEGDNVPVADPYSDGFPVISFGHGRMTTWSAYENIWSVLVQEGYIV...

>7 (GuaPA)

MKRRSKGFSFAIAILLIACITLFFIWGAQNHFYETVLVDTDENPRETHLVGQIDGVFTDENYGEYEVTIYYPAINSGVSQPPDKSGAPYPAIIFAHGWLTS...

>8

MRRAILSWLLLLAVGLGRFIPPDRPGPCAVETGRLSVTTEGGRLPVLIYRPRPPGRLKEQAAVVFGHGLLAPVTAYDSLCRHLASHGFYLLLPDYSSLNTR...

>9

MRFISLISILLLFNLSMAVEIHDLHFYNRYNHTVAATIYLPENLNNSPAVIFCGGWLSSREMYSWLPEELAKRGYVVLAIDPSGNGDSYGKLPSFKIFGVR...

>10

MEKWGVGAILLLGLGTCGSAGCGGGRVTPPDSPGEQMQAELAETLPEGGGREGRESDVAEVDREGESLDQGMDAEQTLPDAIGLTEEVGESGALYEA...

>15

MVMTFPGRNALLTLCLILLVALPAATEAPAGPYNVGYVIYDFPFVSPDGALQSLTTAVWYPTDAEPTPYTYGDGRATGLVSLDGPVSPAGRPFPLVVFSH...

>16

MKKALLLLILSILCVLVYSQVYEIGHTTITFNDPSRSRDIETEIYYPADVAGENVAVSTGTFPVCVFGHGFTMSVDAYENFSDVLVPQGYIILLPSTEDGF...

>17

MKTRGNGTDTQPAARPLRWPLLLCALLLGLLPAACGSDDDSAPGSPAGNEPFYAPDELGPFAVGRSTFTIVDADRGDRELPVDVWYPVDPEEATGAPS...

>18

MKFPQNHYLFVLILLLITLVPVSGCNDGSGGLEKTTELEPEDIYPLGDYYDPGKTGPYEVGVKTILLADNSRRTMLGHTKRILLTEVWYPILAPGDKINTV...

>19

MKFRFYAGILIIAVVLIAGCGKDGGIMTQDVEFTSDDGVLLKGTLYLPAGISEKLPGVTLAHMNMNDRTSWAYYAEKLALEGYVVLAFDLRGWGESGGESD...

>20

MSLVITATMAVGLCGCTDNADTDGADGAEGSATEEGGTEESGKESGEESKADLAALAAEVEEPGPWAVGTTTVETTGARDRQLPVQVWYPVDTAVAATA...

>22

MTMAHHKPRFVLAVLLLCIAAALPAWSVEPLADPAKPGPYPVGVTTMLLVDHSRTDNALAGGPRSLMTEIWYPATDDTRGLPKNRLLDFFNRNTDPGFTVL...

Supplementary Figure 2. Predicted signal peptides for the candidates are bolded and underlined. The underlined sequences were removed before gene synthesis.

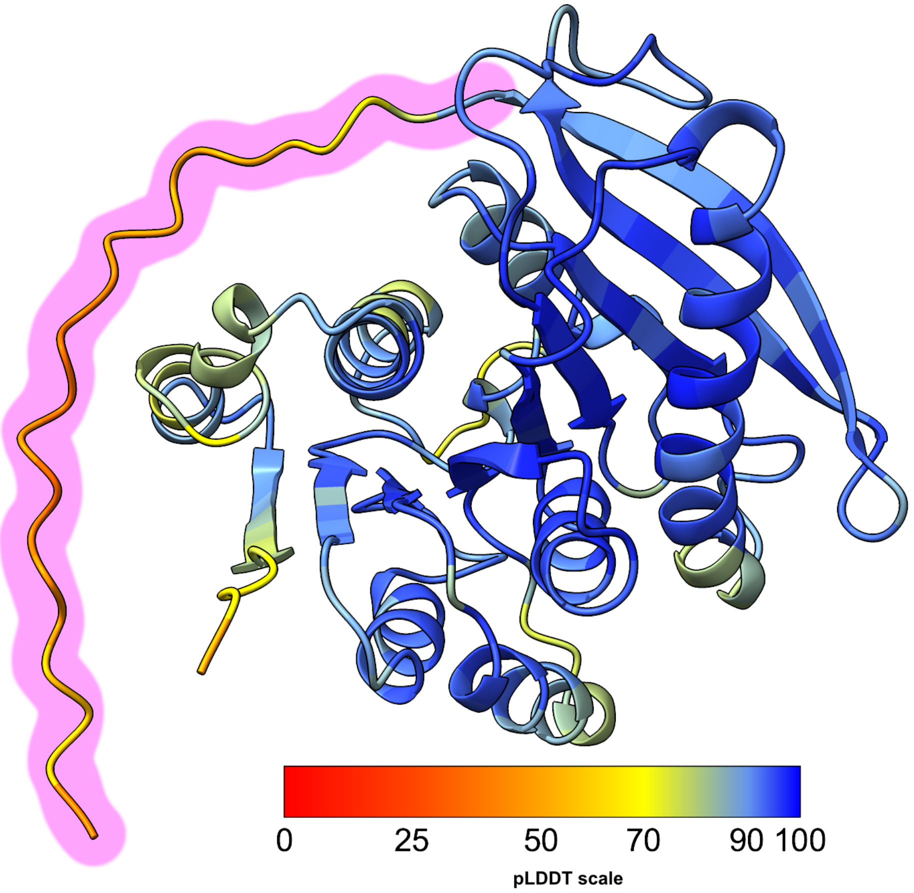

Supplementary Figure 3. GuaPA's predicted protein structure, depicted with pLDDT coloring and the 24 amino acid leader sequence highlighted in pink. The protein structure was predicted with ESMFold and visualized with ChimeraX.

Supplementary Table 1: Amino acid sequence of candidate PETases with original putative signal peptide removal

Candidate	Sequence
1 (B1)	MEKPVVFENEGQQIVGMLHAPDGAEGLSPAVVMFHGFTGTKVEPHRLFVKTARR LAEEGFYVLRFDFRGSGDSEGEFREMTLEGEISDAKASLDFILSQPGVDRGRIGVIG LSMGGGVAACLAGRDERVRAVALWAAVSEDPPDLFQELIKTFEERPDKSVDYVD MGGNLVGKGFFEDLRNVKPLQEISGFEGPVLIVHGDNDQTVSVEHAYRFYERLK GKHPLTALHIIRGADHTFNSHEWEREVIEVTVDFMKRA

2	QSYEMGHRSEIYIDPSRGDRAIPTEIYYPSLTAGDDVPVADPPTGGFPVVAFAHGR<br>MMPWDDYENIWGALAPQGFVVACPTTETGFMPNRTNFSLDLIYVISRIRLENSSA<br>TSPFYGKIGNASAVIGHSFGGVATFMAAIESDSITAIVTFASGQIDSVELAGISEPALI<br>FAGTDDSLTPAATFQIPYYNNLGSSCKTLVNIIGGTHCHFAESSYYCDIGEAGFPPP<br>GITRERQHELTNQFLIPWLDFMLNTDPVASDSFQTLLTTSGDILYLQDCDIMGVSR<br>DDPTDLPGIIDLLAYPNPFNSAVRIGVERGEWRVENREIFDITGRMVAEIPAQSVIP<br>DPDRESRGVVEGLDSRFHG
3	VGTISFDLVDKERLELYGDKIGEERKFKAQMWYPSDDVTEGERAPWIEDGVKVS<br>RMIPKDIGLPGFLIDYSSLIKSNSYKGVPISEKEEKYPIVIISHGWTGYRNIHTDIGEL<br>LASHGYIAISIDHTYGSLATVFDNGEVIEVDYGALPNRNETEDFLDYANALVTTYA<br>YDSRFVLDYLEELDDSELIDGRLDLDRIGTLGHSTGGGGVVKIAIEDDRIKAIFGFD<br>PWVEPIEDELLKIGLDVPSLFIRSEQWEEGYNNGYLTTLINHSVIKPMTYQMNGVN<br>HLDFTMLYMYRPILQAFGFAGELDSQVSSKIQLEYVLKFFETYLKDNETNLNDIEK<br>IYDSVIPVNY
4 (B4)	QNSANVQNEISYDVFDYAQHPMGTIWGDPLPDAPALAARGEYMVGVQTLHLSNP<br>NQTDVINTITSKSTVIYDRPLTVEVWYPATLKAGQVQVTVYCDFLGRYDKPGTLV<br>PYTFLGRATRDAVPDTADGPYPLIIISHGYPGSRHLLFYLGENLASKGYVVVSIDH<br>TDSTYKDVGHFASTLVNRSLDQKFVIREILKMPIWKDLCNPNEVGIIGYSMGGYG<br>ALRTLGAGLEDSNTIKGFLGEFASMLIAKPSDEGDPLVKAAVLFAPWGGSFGTGP<br>TGLWDKASLAKITVPTLWIAGSQDDIAGYEGIVNLFNSSVNSRRYLLTYDNALHN<br>VAPHPAPSIATEFGDYYRFADPVWDMWRIDSINEHFVTAFFDLYLKGDESAGSYL<br>DMKVENSNEGVYSDQAESSTYWPGFQPRTALGLKFR
5	MKIIRSKVCCQRGKVKIQAIQYRPETNFAFPGVIILHGIEGFKAHHEEFGERIAKEG<br>YVTLIPQWFGGETEGKNPDEVELNDIFSIVESLRSSKYVDKTRIGLIGFSLGAALALI<br>FASSNKDIKALILYYPPADREKITQFFKKIRISSDFIKNILSPTLIIQGDNDRIVPIESTC<br>KLLQEFKCYNKLCEMRVYPDADHAFNWPDRGEYNAEAAEKAWRDMIEFLNRYL<br>TPNPVDFPGR
6	QSYQIGHTSIIYYDANRSNREIPTEIYYPAENEGDNVPVADPYSDGFPVISFGHGRM<br>TTWSAYENIWSVLVQEGYIVAFACTEMSFIPNHTEFGLDIAFLVNRIQAEGIDSTSL<br>FHGKIGTTSAVMGHSMGGATCFLSAQGNETITAITSFVSGNTSPSPIDAAVDITIPA<br>LLFSGSDDYMTPAEEYQIPIYEALNSDCKTLISITGGNHCQFAEYDYYCDTMEDNY<br>PEPALTREEQHEVTNRFFISWVNFNLKGDFGAWYEFEDSLMTSEAITYMHDCDVT<br>SVLEEPSETNIFMEYGLSNHPNPFNLTTTIKYELPKSAHISLKIYNISSKLIKTLVDEK<br>ENTGYNSVIWDGKDENGNPVPSGIYLYNIEIDGRVFSETKRMILLK
7 (GuaPA)	WGAQNHFYETVLVDTDENPRETHLVGQIDGVFTDENYGEYEVTIYYPAINSGVSQ<br>PPDKSGAPYPAIIFAHGWLTSKELYTWIGNYCAAQGYVTLLFSVPDPTSLEAFRQS<br>VTGITKSIDYLLVQNQGGLLSGLINTSRIGVMGHSMGAMAVLIATTEDSRIKAAVS<br>LAPGYFGSTTKKYVEACKSIRVPIQFQAGSLDKICPPSAVETYYNAVRIPPKEIIVIN<br>GADHIQFSDAPATLWANITLEEQHETSRKYFIAWFNYYLRDDFNYYAYLFGSEAR<br>KDMENGILSSLEYVERFDC

8	GPCAVETGRLSVTTEGGRLPVLIYRPRPPGRLKEQAAVVFGHGLLAPVTAYDSLC RHLASHGFYLLLPDYSSLNTRRERIPRILLRVVDLLDSLAQAPGYRLDPKNVALVG HSLGGGAAFWAARLDTLQRVSAVCGLAPFPYAKEIMPESLRVPVLLLVGEKDWT ARPDMVRSFFYLPCSTFKQLVRIKKGAHNGFLDSPVRFPGFDHRSQLRAARRYLV AFLRFYLDGDFRCASYLFGDFAEADTTVAIEHHPPGFGAVNLILWVYPNPCRSRV YFRSPSHSLDSCQIFRTGGHYIRTITPADSPRMFWDCTDSAGRPVPDGRYVVHGFT ATQLFYLEKEPFLIRRH
9	VEIHDLHFYNRYNHTVAATIYLPENLNNSPAVIFCGGWLSSREMYSWLPEELAKR GYVVLAIDPSGNGDSYGKLPSFKIFGVRIFSVIDGPYNYINGVWSSDIYDAVTFLTT NYRVSKNVALIGHSMGGFAVTDEALKDGRIKTVIALSDADIYAIKNLKIPVMVITG DFDLIINDNLVAIPGYNAANPPKELIVIKFGTHNGFTNLIWPMPPWNHNVTLYYLS SWLDCFLREDRESCKTLEKPHKYLSTLSESKCNLDGKEKVIKRATGRRKTFTAFLL IFLFLAVLILYLRSKEG
10	CGGGRVTPPDSPGEQMQAELAETLPEGGGREGRESDVAEVDREGESLDQGMDAE QTLPDAIGLTEEVGESGALYEAEGEAETIDGCDLADQGGEIEAWETLPPADVQQD EQLAAGTGDPFEWGPYEIGKKDYDFYDIQRMRLVPTTVWYPALPSGQSKAKYLL VVQGKAYVEPPAEPSGAPYPLVLFSHGYGGTRFQSVSYTEYLASHGYVVAAMDH IGNTFLDFFSDDAKVAQIALERPKDVRFVYQELVKLSNSGDGVMGGMVDQDRVA VTGHSFGGYTALVVAGAEIDVSEAIAACQSGAVSDIFCAYVEYWPAGEVIKLDPPI PGVQAAIYLAPGGYSAFGDQGLAAVKVPSMIFGGTLDDTCSVAVEIEPIYQALPPP KIEVLIQNGGHMSFTNLCDLPFAQFYLDKYCDVEGMIPAQQAFAVTNGLSVAFLD LYLKGVDAAAQYLTQEAVDEEFGYVELKAE
11	MMKDIAITFREGTIAARIYLPPPVHRAGEPHPGIIILHGYLAQQIQFDDLPRRLARK GYVAITFDMPGHGRSSGKRGYIEKDLQIDAATTVLDHLISLPNVDPKRILVIGHSL GGAMGTYLCARDPRPTAFVAIAPAGSLKRIVPLPARMLFGIPYIIHRIKKRMTGNS LYFHSIVTEHIVWADKRKVKFELERELLQHRQPVDNYRTIFLIDPRIPARKVTIPTI MFLASRDRVVPNRISRELFDNIASDDKTLLTIENSGHSIMSDWKKEDFWKMLVPW LETHIPP
12	MSQYDPFLRGPFPAGVKTYEFEDTSRDRKLPIECWYPATSAYKGKDLDKETQDQF QLMEGFPKARQNAVRDAEIVDDSFPLIIFSHGFAGHRRQTTHFCTHLAGHGYIVAS IDHIGSTLPEIMQMVMKVRKEGKLPDIEKFMEDVANDRPVDATFTIDTILAGELGP GIDPDRIGITGHSFGGWTTLVTTKSDTRIKAALPLAPGGGKSPLYGKDNPLTKMLT LDWERTVPTLYLTAEFDTLLPLDGMKDMFDRTPEPKGMVVLLNADHFHFCDGVE VVHDFFRQMGGFVGGMDPGGESEPAPMKKAKPSSELCPGKDAYTFIQGIGLAHM DAHLKGNSDAIELLKGDIAALLADRGVKVEVMQG
13	SRTRQILTEIYYPADTAGEEVPVAEPGAEGFPAVSFAHGRRMAWSAYSNFWEML APQGYIIVFPCTEMGLSPNHLDFALDCLFDAHKLQELNDLGTSIFYQKVGDTFAVT GHSMGGVVSFIAAIEDPTVTAIANFACGQASSYDLASVTAPALIFAGSDDYMTPV DQYQLLFYNELSSTCKALLILQGANHCQYAQYTSVCDDIEDQYPDPALTRTQQHA VTSTLLIPWLDYMLKGDEEAWTEFNVLAGDGDVTETHMICDLPFNTPTPASPPTP FVPATSSGGIGTLIVVISGTLALLSSRRK

14	MSRAFSFASVAALLVFFAASIWLARLDKGGPPHTELMLEGEVPATLYLPGDGEHR EAFLDAPPRDERPPAVVMMHGFSGDRLSMSGISRRLAESGYAVLNIDARGHGQN RNPFSSSWAAPDLFYPDLLAAVEFLRAYPFVDGSRIAVMGHSMGAGASLDYASR DSAIGAAVLVSGGWRIQGPHRPPNTLFLYATGDPERIRGRSRQLAAGLAGVNDPE VGRTYGDPTRGDAVRLAEVAGADHQTIVWREAAVRETVAWLDATFGVARSPGP VPDDPRAPLLPLIWAAFLLVMVGLGQVVGRLVPAQPYRPGGGRIAGLAAIAAAFA LTMPLLALGTPGFILSMEVGDVFVSHFALAGIVLLVAIRMREPELIDSLFAPRLLPT LIGAAVGTIGVFTLMRVFEPVMHRLTLTPERFLVFVMAALGLLPLALAFNLLLRR GTTAGATLTSLAGRVLVLLVLFAGVQVGILSMVMLFMLPALGFVCLQFEVLAAAI YASSRNLLALSLIDAAWLAMVIAAIMPIRI
15	APAGPYNVGYVIYDFPFVSPDGALQSLTTAVWYPTDAEPTPYTYGDGRATGLVSL DGPVSPAGRPFPLVVFSHGYGGGGTANLFLTKHLASWGFVVASPDYPDPDQMVR IRGGGTNTPILQYLRNALRLARTGLDFPFDDYAFRPAGLRTVIDQMLAQNADDTS ALHGAIDPDRIAVAGHSLGTFTALSVAGLDPQWTDPRIRAVLALSGGVFMWPTQ DFAQVSVPIMFMYGEREAGQRERIGLADVAALTRAAYDVCQPPKFMLEVKGGTH FTFGQRVYEERRAGGDASLPERQVAVINSYAAAFLRLYLRNDTSAAATLQQTDP MLTLYRHDM
16	QVYEIGHTTITFNDPSRSRDIETEIYYPADVAGENVAVSTGTFPVCVFGHGFTMSV DAYENFSDVLVPQGYIILLPSTEDGFSPNHDEFGLDLAFLVDEFQLENTNGTSLFY GAIAPETALMGHSMGGGCSFLAAESNSSVTTLVNFAAAETNTSAIAAAANITIPAV VFSGEVDPVAPPADHQIPMYNALASECKTYISINDGGHCYFANYNFYCTMGEEA MMPGGPPLTREEQQQTTLDFLSIWLDYTLKGNANALTVFNDSLASSPRITYQQDC NLTILDQINPNAEFKVYPNPADQFFILESSREIKHVLIYNSMGSICLERNKRHRIDISE LPEGMYVIKAITESGSFTKKIMVLH
17	CGSDDDSAPGSPAGNEPFYAPDELGPFAVGRSTFTIVDADRGDRELPVDVWYPVD PEEATGAPSLYQVSILVWIFPLVFTTPSEVALDSPPVSEARRFPLIVFSHGSGGLRYQ SFFLTEILASHGFVVVAPSHVGNTLLDEINGTFAPLTDMMVARPLDVSYLITRMLE KNEDAADPLYGTIDADRIGVCGHSFGGFTSLAMAAGFGADPPPDLDPEIPEDLEPV PPDPRVDAIVPIAPASSWFGDSELAGIDVPTLIIGGTLDTTTPIATENVRPYEMIPAP VFRADLDGAVHFSFSNSCDLIQGMREKGIPQALIDALLGAEFTAPCNPPSLDVEEA HRITNLYTVSLFETFLEDDSRYEKYLTEAYAEASEPNVTFYANPD
18	CNDGSGGLEKTTELEPEDIYPLGDYYDPGKTGPYEVGVKTILLADNSRRTMLGHT KRILLTEVWYPILAPGDKINTVGDMIGQLPEWTPQILESFYGENWEDLLNIQTSAW RDAKTFLPDHPFPVIFFSHGLTALRFQNYTLCEHLASHGFLVIAPDHFDNNVFSNIP GHFTWFNPISTVASEFQRPRDIAFLVKHLQTRQSQTATFFGSYADPGRLGITGHSY GGMTSYAAGEQVAEIDAIAPLNPVILVPTTKFFTKPLLLLVGENDNLASSMFNSCE VAKRNYQTHKGNKAFLFLKNSGHYSATDACMLLPPGFINDDITGCGGAMLNPDI ANEIVAAYQVAFFSITLKQDARYQDYLNKNNYPDNLDYEVSWK
19	CGKDGGIMTQDVEFTSDDGVLLKGTLYLPAGISEKLPGVTLAHMNMNDRTSWA YYAEKLALEGYVVLAFDLRGWGESGGESDFHEMYRDVMAAVTYLANFGKVDR NRIASAGASMGGMASVIAASRTSSIKAVATVSSPPAWSESEPVKVIGKLPPRPVLVI AGSSDPHLDLRAARMLFLAAKEPRQWLEIKTNKHGTDIFATPQGVELERALAMFF AENLKDSGRSSTTEKDGK

20	CTDNADTDGADGAEGSATEEGGTEESGKESGEESKADLAALAAEVEEPGPWAVG TTTVETTGARDRQLPVQVWYPVDTAVAATAEPATYDFPGIEVPAGAVTGASPAP GPFPLVIYSHGNGGLRYVSSFLAEHMASHGFVVMAPDHVGNTALDTFLGSRDET DQVAEDRPVDVQAVIDAATSGQAGLEEVSPVVDGEEVAVIGHSFGGYTALALAS RQAEGDDSLDAIVGLAPASSGVDDTTLEAVGVPTLLISGTLDETTPIEEDTLRPAEL VSGRPLVRADIDGAGHQSFTDVCDYLGIAESQPDLPAALVEAPQEYALEGCAPELI VIDEAQRITNRLVTAFLLETLYADDSWSPLLSPGAEEGQPLALLERED
21	MTKVNFQNKEEKRIVGILSIPKGKGPYPAVIFCHGLGSSKESPLIKTVSKKVRQVGF ATFTFDCLGSRESDPYHSTAGITKRLSDLDAAIDFLAKHPRVDAERMGLYGHSLG GSVALVQASRDKRIKTAVLISPVIDFRRTGLRPINKKHMDEWDKREEILTINRKNKI VFRIPWDFFVDITSYDFVDIARKVACPVMIIHGTQDSLVPIQQSQILCQNLKNSELIS LPWQKHFTRTTRKRKEAIVNSIFWFNKYLKKSATPSN
22	VEPLADPAKPGPYPVGVTTMLLVDHSRTDNALAGGPRSLMTEIWYPATDDTRGL PKNRLLDFFNRNTDPGFTVLLKMAFGIDLIQADKDFRNDAVRDARVRDGRFPLVL FSHGNGGIRMQNAYWCEHMASHGYIVMAPDHTGNCAVTFIDGRIFPFNDSEEGR EQSRQDRPKDISFLIDAMERMNKGADSRFMGKVDLDHIGVAGHSFGGFTSTWVA DAEPRVDAIVPMAGAGAERVNYTCPVMVLVATEDDTLGPERMADLRRYYDESK GPRYLVEFLNAGHFSFTEMYQLKPDFGDGCGTGTRITNGEPLTYIAMDVAFPLIKG YTTAFFGKYLKGLDGYDAYLAANHNPNELVVKSSIPETPQ

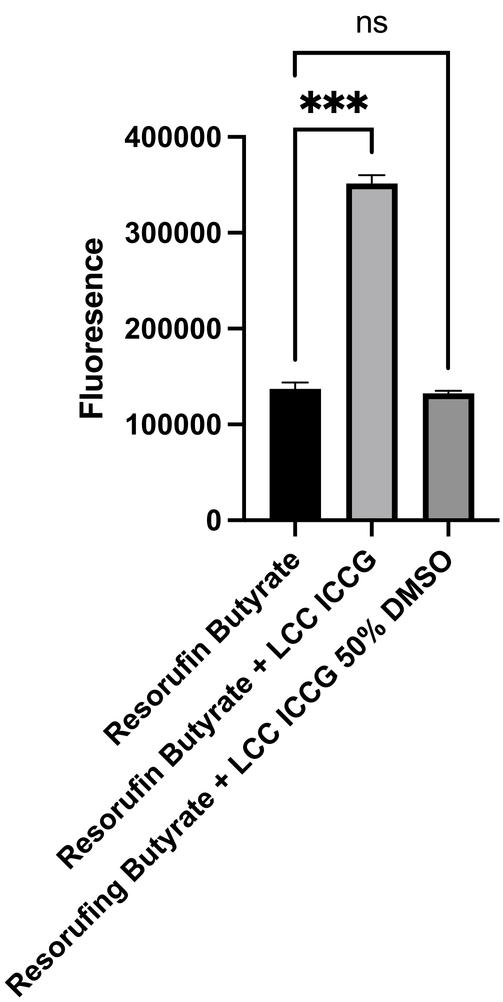

Supplementary Figure 4. Inactivation of PETases via DMSO was verified via the addition of DMSO to a final concentration of 50% v/v and adding resorufin butyrate 10nM and incubated for 5 minutes at 37 degrees. The fluorescence was the measure and compared to a no enzyme control containing only resorufin butyrate. The fluorescence was measured utilizing excitation wavelength 571nm and measuring the emission at 584nm.

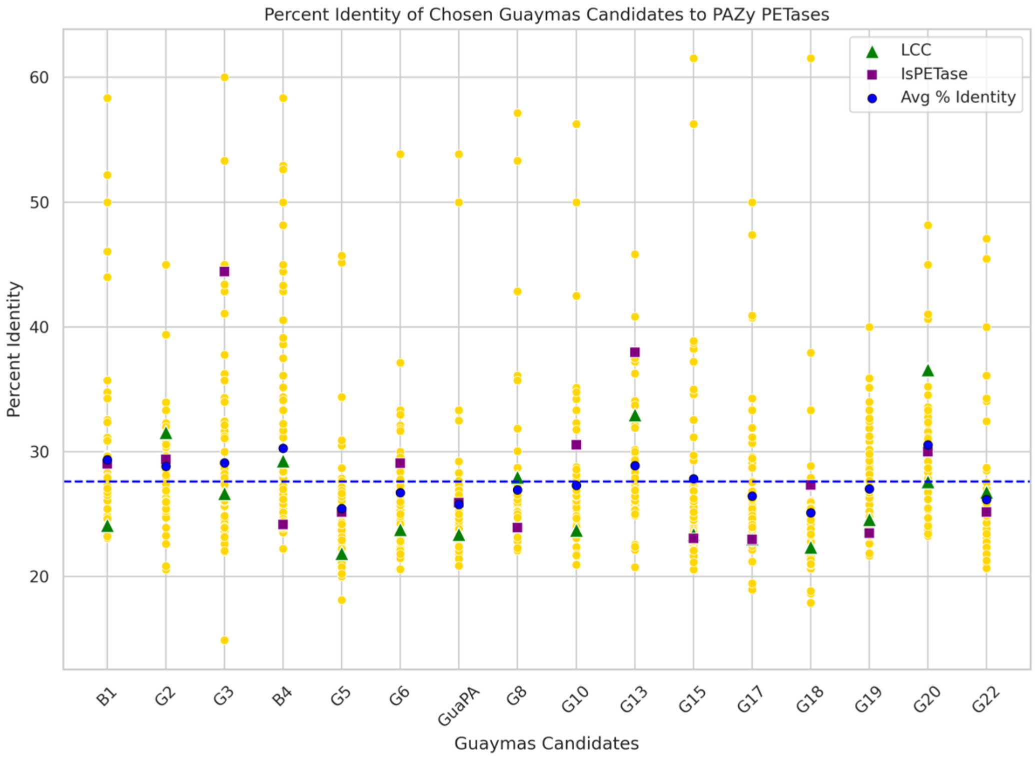

Supplementary Figure 5. The percent identity of chosen Guaymas candidates to PETases contained in the PAZy database is depicted. The sequence identity to Leaf Compost Cutinase (LCC) and IsPETase, two canonical PETases, are highlighted with a green triangle and purple square, respectively, for each candidate. The average percent identity for each candidate is shown with a blue circle and the overall average identity of 27.6% across all of the candidates is denoted with the blue dashed line.

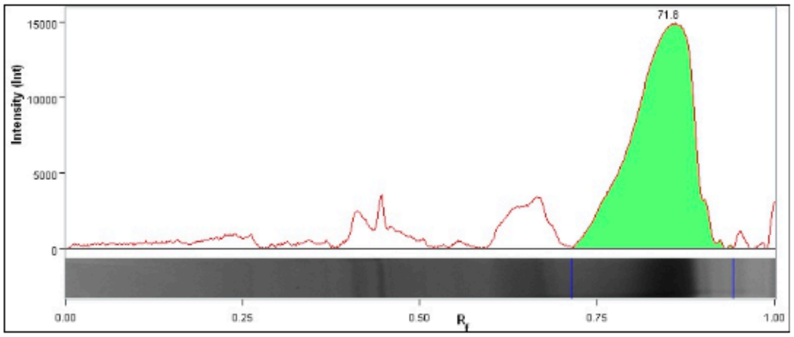

Band No.	Band Label	Mol. Wt. (KDa)	Relative Front	Volume (Int)	Abs. Quant.	Rel. Quant.	Band %	Lane %
1		N/A	0.851	92,035,772	N/A	N/A	100.0	71.8

Lane Background	Lane background subtracted with disk size: 10
Lane Width	6.28 mm

Supplementary Figure 6. Example quantification of protein purity via SDS-PAGE using an aliquot of crude LCC-ICCG. Lanes were determined using image lab (BioRad) and quantified using the lane profile tool under the "lane%". This gives the percent of the stain in the sample contained within the band of interest.

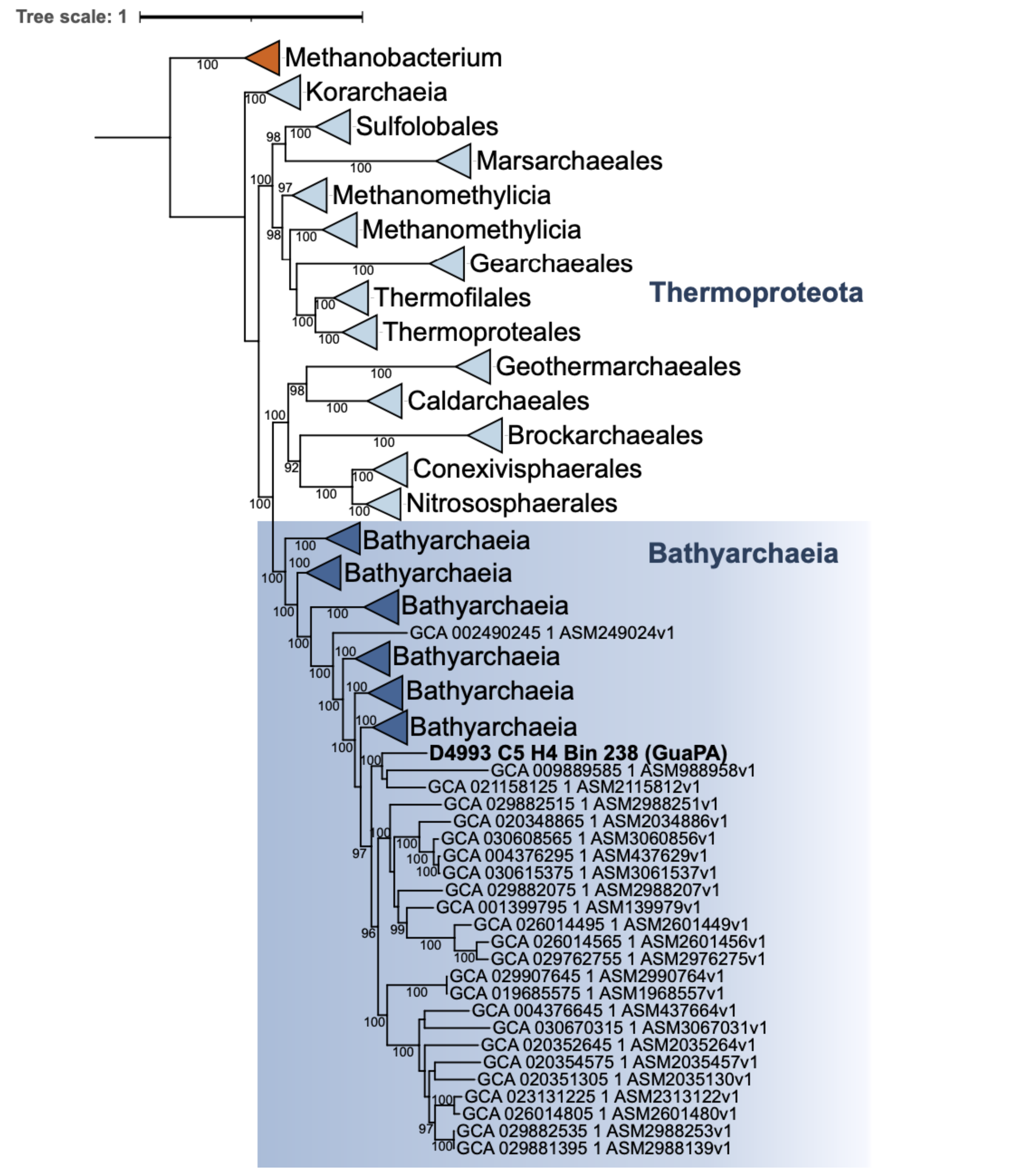

Supplementary Figure 7. Phylogenetic placement of the GuaPA-encoding archaeal genome within Thermoproteota (previously the TACK superphylum). Maximum likelihood phylogeny, using Phylosift 37 markers and inferred with IQ-TREE multicore version 2.0.7 and the best-fit model LG+F+R10 chosen according to Bayesian information criterion (BIC). The analysis includes 41 Methanobacterium (orange) and 1067 Thermoproteota (blue) reference genomes, rooting with Methanobacterium. The shaded regions highlight the class Bathyarchaeia. Ultrafast bootstrap support values >= 90 are shown.

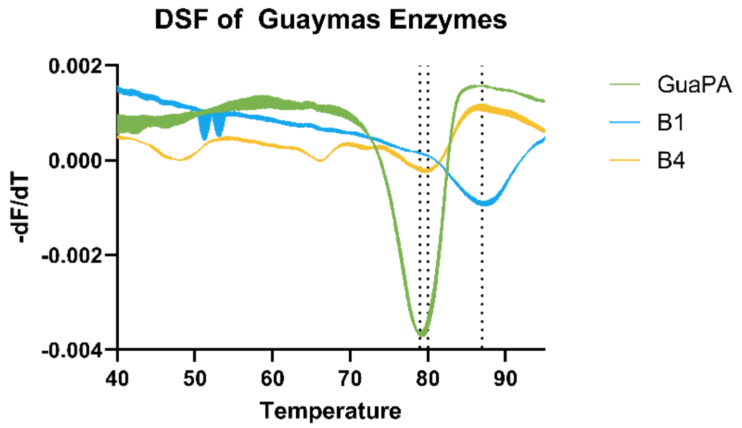

Supplementary Figure 8. DSF of GuaPA, B1, and B4. The melting temperature of all 3 proteins was calculated using the GloMelt kit by Biotium and the curves are representative of the change in fluorescence of triplicate samples analyzed using a roche light cycler 96 and the corresponding software. GuaPA has a melting temperature of 79 degrees while B1 and B4 have melting temperatures of 87, and 80 degrees respectively.

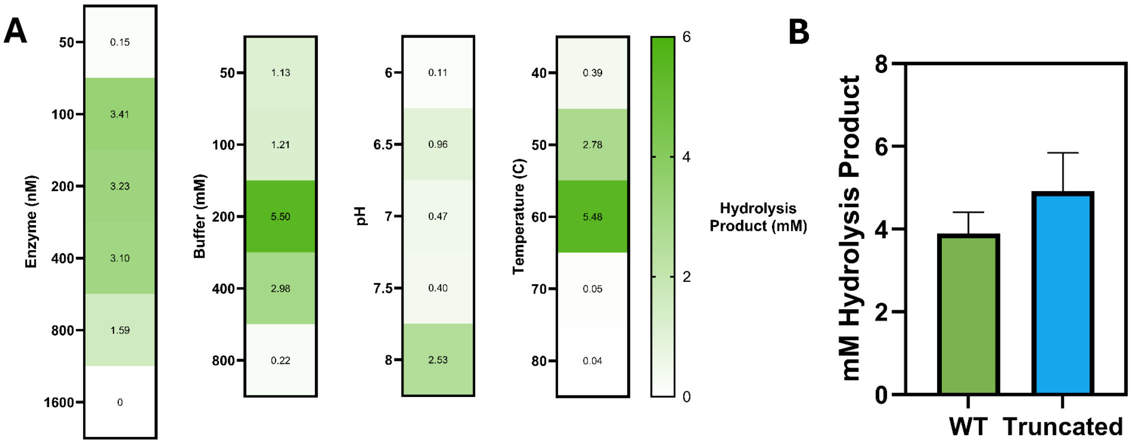

Supplementary Figure 9. Impact of reaction parameters on hydrolysis of PET film by GuaPA. **(A)** PET film hydrolysis as a function of enzyme concentration, buffer concentration, pH, and temperature. For all reactions where they are not the variable being tested the conditions were 200nM of enzyme, 100mM KPB, pH 8, and 60 ℃. The heatmaps represent the mean of triplicate experiments. **(B)** PET hydrolysis using optimal conditions using after removing a 24 amino acid signal peptide seen in the predicted structure of GuaPA.

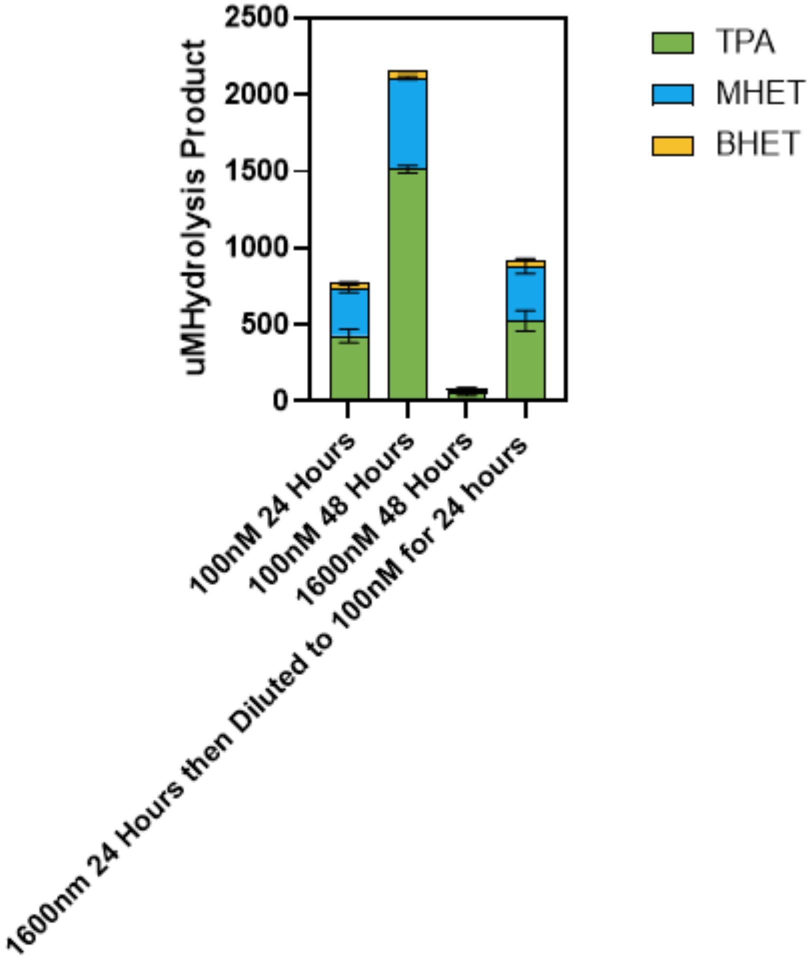

Supplementary Figure 10. Impact of enzyme concentration on GuaPA activity. GuaPA functions optimally at 100nM. After 24 hours GuaPA can hydrolyze 777.424 µM of combined BHET, MHET, and TPA. and releases 2152 µM after 48 hours. After 24 hours at 1600nM only 63 µM of TPA is released by diluting that reaction to 100nM and allowing it to continue for another 24 hours the released product is almost identical to a standard 24 hour enzyme reaction releasing approximately 900 µM of product.

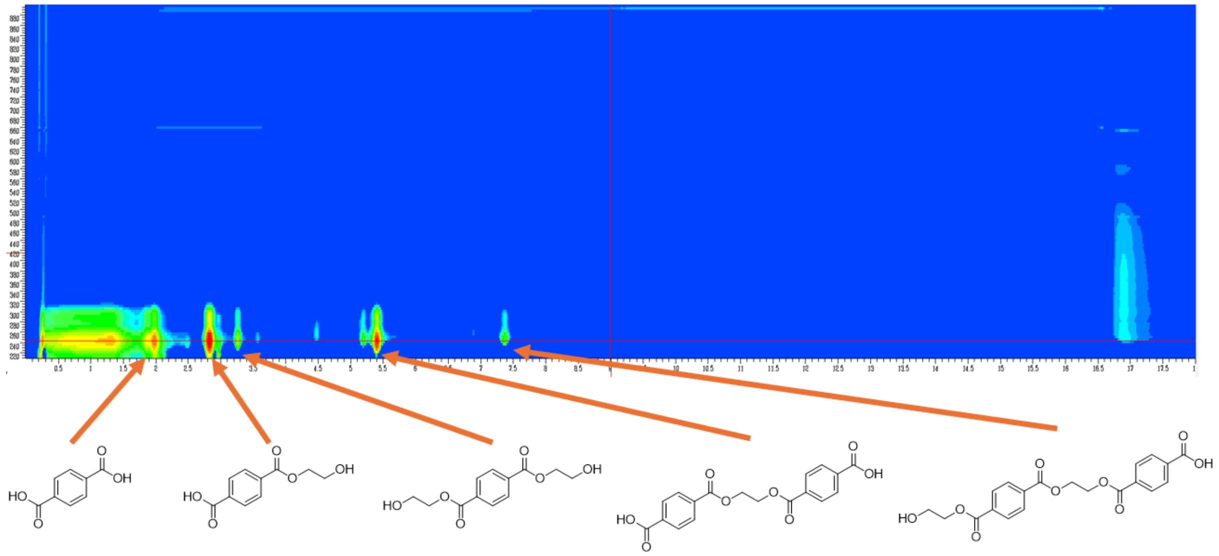

Supplementary Figure 11. LCMS total spectra of degradation products of GF PET by GuaPA and identity of corresponding compounds.

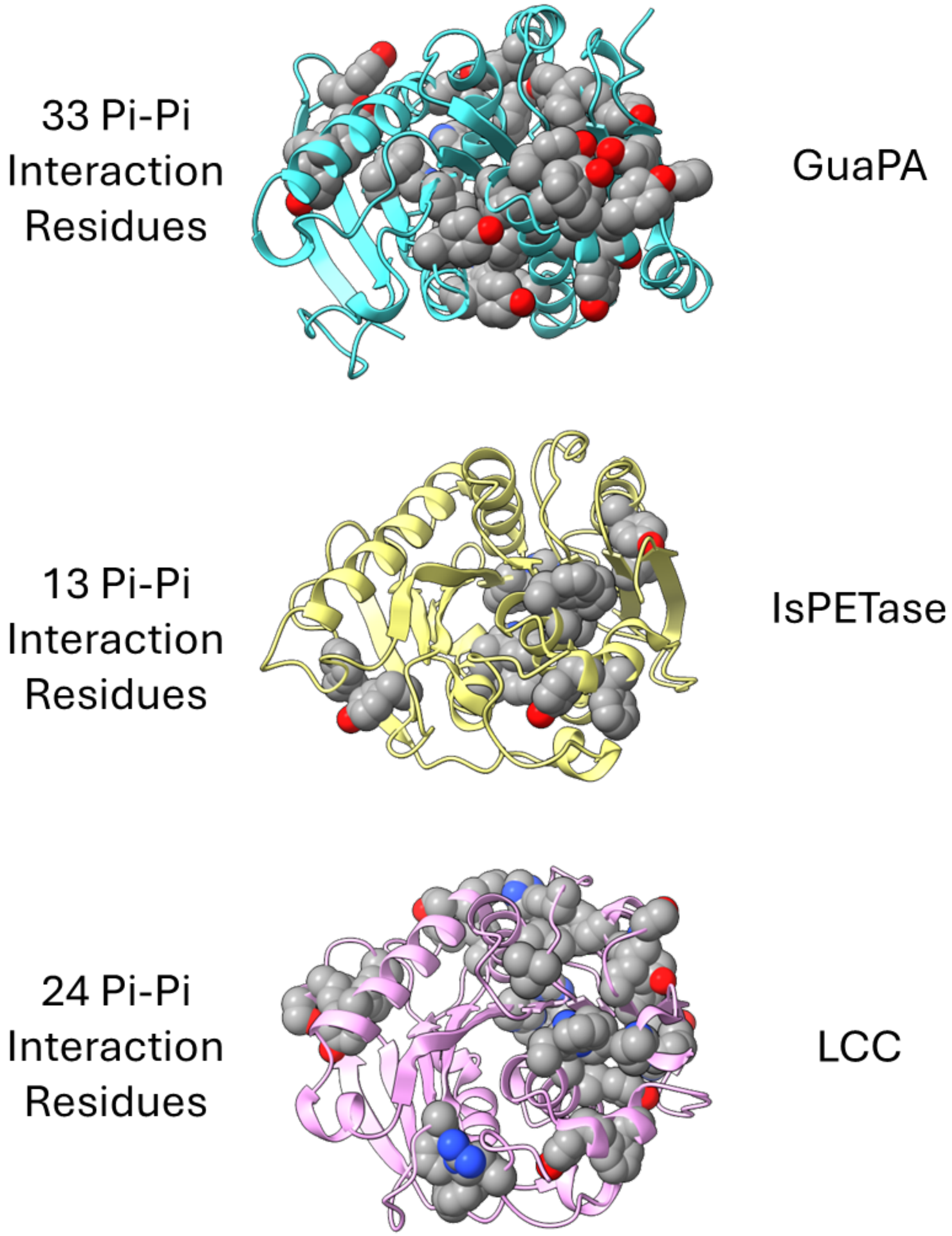

Supplementary Figure 12. Structures of GuaPA, IsPETas, and LCC with residues predicted by contacts of aromatic residues in Proteins (CARP) are shown as space filling models.

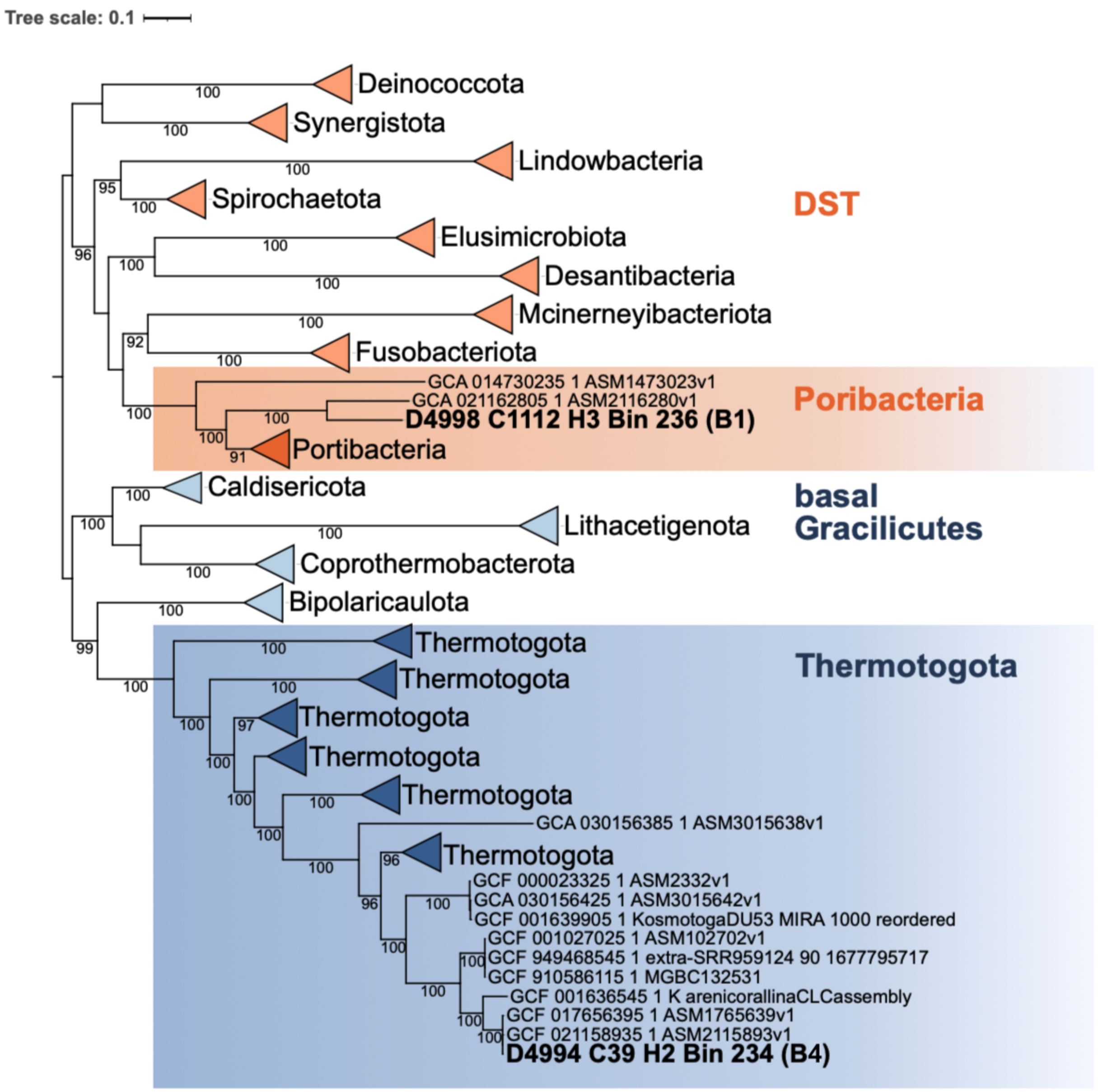

Supplementary Figure 13. Phylogenetic placement of the bacterial genomes encoding B1 and B4 within the DST and basal Gracilicutes. Maximum likelihood phylogeny, using Phylosift 37 markers and inferred with IQ-TREE multicore version 2.0.7 and the best-fit model LG+R10 chosen according to Bayesian information criterion (BIC). The analysis includes 294 DST (orange) and 356 basal Gracilicutes reference genomes (orange), rooting between the two clades. The shaded regions highlight the phylum taxonomic levels of each PETase candidate-encoding MAG. Ultrafast bootstrap support values >= 90 are shown.

Supplementary Table 2: CARP analysis of GuaPA

Residue 1	Residue 2	Distance (Å)	alpha angle	beta angle	Shift
A 8 PHE	A 16 TYR	4.9126	16.0147	58.86	4.102
A 8 PHE	A 73 PHE	6.8873	55.6021	17.9981	6.0133
A 8 PHE	A 97 TYR	5.903	44.7034	131.3812	4.9211
A 13 TYR	A 16 TYR	6.9319	93.4624	1.059	6.2164
A 16 TYR	A 73 PHE	6.3183	117.8764	3.4739	0.7576
A 45 PHE	A 56 TYR	4.6771	45.625	9.1468	3.8638
A 47 HIS	A 56 TYR	6.854	27.0257	72.0604	6.3351
A 47 HIS	A 73 PHE	6.4794	54.3064	104.732	3.8676
A 49 TRP	A 84 PHE	6.7784	28.7184	64.4439	4.7472
A 56 TYR	A 58 TRP	6.9557	36.0058	49.0799	6.2817
A 56 TYR	A 121 HIS	5.5371	56.0025	11.7462	5.2957
A 58 TRP	A 225 HIS	3.9654	16.364	115.1315	0.4928
A 62 TYR	A 232 PHE	5.8642	91.7579	141.7408	4.4069
A 68 TYR	A 236 PHE	6.2714	55.1557	140.0922	3.1697

A 84 PHE	A 149 PHE	5.7712	74.2106	54.4236	4.5495
A 84 PHE	A 156 TYR	6.243	50.497	116.4378	5.0094
A 121 HIS	A 205 HIS	4.6391	39.2888	164.1042	3.0389
A 121 HIS	A 208 PHE	5.5714	40.0492	85.8333	4.4337
A 156 TYR	A 186 TYR	6.8232	95.9353	33.1155	4.2944
A 169 PHE	A 186 TYR	6.8867	5.3488	75.8193	6.1216
A 169 PHE	A 187 TYR	6.4495	93.0731	109.9853	5.6668
A 231 TYR	A 249 TYR	6.7353	83.6703	96.9089	3.4748
A 232 PHE	A 236 PHE	6.6074	57.8161	113.3993	6.1362
A 235 TRP	A 239 TYR	4.5015	56.1733	43.3167	3.0032
A 238 TYR	A 239 TYR	4.84	76.2145	30.3308	3.399
A 238 TYR	A 244 PHE	5.1844	100.4519	107.2902	2.68
A 238 TYR	A 247 TYR	6.2637	41.6279	22.1616	5.6191
A 246 TYR	A 249 TYR	6.067	35.462	4.3249	5.6026
A 247 TYR	A 269 TYR	6.212	106.6972	89.4548	2.9276

A 251 PHE	A 269 TYR	6.5803	33.2562	153.2174	6.0899

Supplementary Table 3: CARP Analysis of IsPETase

Residue 1	Residue 2	Distance (Å)	alpha angle	beta angle	Shift
A 26 PHE	A 41 TYR	6.1697	58.1248	170.7435	5.8601
A 67 TRP	A 68 TRP	4.2572	28.5824	3.534	1.4659
A 68 TRP	A 172 PHE	6.3091	37.4811	93.8289	5.5322
A 130 TRP	A 208 HIS	4.6898	40.6024	137.1484	3.3566
A 190 TYR	A 200 PHE	5.4222	47.3679	147.0418	3.3243
A 228 TRP	A 232 PHE	4.5753	53.5782	39.6102	3.0246
A 239 TYR	A 242 PHE	6.6191	56.3541	7.0596	5.2261

Supplementary Table 4: CARP Analysis of LCC

Residue 1	Residue 2	Distance (Å)	alpha angle	beta angle	Shift
A 56 PHE	A 86 PHE	5.0688	76.077	61.5106	1.9502
A 71 PHE	A 141 TYR	5.7801	109.7729	63.2529	3.6009

A 95 TYR	A 125 PHE	5.1387	32.6723	70.1275	4.0013
A 95 TYR	A 127 TYR	5.9272	81.8919	59.4305	3.0534
A 127 TYR	A 190 TRP	6.5266	91.4942	71.7155	2.3412
A 164 HIS	A 242 HIS	4.9121	23.5965	176.6798	3.2963
A 190 TRP	A 218 HIS	4.8025	26.2819	20.8465	3.7194
A 191 HIS	A 196 PHE	5.31	58.3736	83.02	4.5505
A 191 HIS	A 222 PHE	6.9605	83.0005	95.6063	4.3895
A 196 PHE	A 222 PHE	5.7959	100.5862	5.2578	2.7194
A 218 HIS	A 222 PHE	5.9669	98.9525	13.4836	4.6647
A 223 TYR	A 234 TYR	6.4065	37.1583	146.1788	4.5446
A 242 HIS	A 243 PHE	6.7592	64.165	100.0829	5.0208
A 255 TYR	A 285 PHE	6.6138	39.7815	127.3467	5.7261
A 259 TRP	A 263 TRP	4.9434	67.2108	16.6243	3.4913
A 259 TRP	A 291 HIS	6.4406	81.5698	73.2166	4.2237
A 263 TRP	A 291 HIS	6.6705	60.5557	85.8522	5.4259

A	270	TYR	A	273	PHE	5.9125	61.3611	9.0118	5.2303
